# Impact of chronic psychological stress on platelet membrane fatty acid composition in a rat model of type 1 diabetes Mellitus

**DOI:** 10.1186/s12944-024-02067-3

**Published:** 2024-03-08

**Authors:** Inga Bikulčienė, Justinas Baleišis, Eglė Mazgelytė, Romualdas Rudys, Rūta Vosyliūtė, Renata Šimkūnaitė-Rizgelienė, Arvydas Kaminskas, Dovilė Karčiauskaitė

**Affiliations:** 1 Department of Physiology, Biochemistry, Microbiology and Laboratory Medicine, Institute of Biomedical Sciences, Faculty of Medicine, Vilnius University, 21 M. K. Čiurlionio St, Vilnius, LT-03101 Lithuania; 2 Department of preclinical research, Centre for Innovative Medicine, 5 Santariškių St, Vilnius, LT-08406 Lithuania; 3 Department of Anatomy, Histology and Anthropology, Institute of Biomedical Sciences, Faculty of Medicine, Vilnius University, 21 M. K. Čiurlionio St, Vilnius, LT-03101 Lithuania

**Keywords:** Chronic stress, Platelet membrane, Fatty acids, Diabetes mellitus

## Abstract

**Background:**

Chronic stress and diabetes mellitus are highly associated with oxidative stress and inflammation, resulting in cell membrane disruption and platelet activity. This study aims to evaluate the impact of chronic psychological stress on the composition of the platelet phospholipid membrane and platelet activation in type 1 diabetes mellitus (T1DM).

**Methods:**

We enrolled 35 mature healthy female Wistar rats and randomly divided them into 4 groups, namely the control group (*n* = 9), stress group (*n* = 10), T1DM group (*n* = 8), and T1DM + Stress group (*n* = 8). The Wistar rats were treated in different experimental conditions for 28 days while being provided free access to feed and water. The concentration of corticosterone in blood serum and hair samples was measured using a competitive enzyme-linked immunosorbent assay. Gas chromatography-mass spectrometry was conducted to identify the methyl esters of fatty acids (FAs) in the platelet phospholipid membrane. A quantitative determination of 11-dehydro-thromboxane B2 in the blood serum was also performed using a competitive enzyme-linked immunosorbent assay.

**Results:**

After 28 days, the concentration of corticosterone in blood serum (ng/mL) was observed to be higher in the stress group as compared to the T1DM and T1DM + Stress groups (*P* = 0.031 and *P* = 0.008, respectively). The percentage of C 16:0 FA in the platelet membrane was greater in the T1DM + Stress group, but its levels of C 20:1 omega (ω) 9 FA, including C 18:3ω3 FA, C 20:5ω3 FA, and the total sum of ω3 FAs, were lower as compared to the control group (*P* = 0.016; *P* = 0.016; *P* = 0.031; *P* = 0.016, *P* = 0.031). The concentration of 11-dehydro-thromboxane B2 in blood serum (pg/mL) was observed to be higher in the stress group than in rats with T1DM (*P* = 0.063).

**Conclusion:**

Chronic psychological stress is related to higher levels of corticosterone, saturated FAs acids in the platelet membrane, and greater platelet activation. This study proves how a low percentage of unsaturated fatty acids in the DM and stress groups indicates the disturbing impact of the oxidative/inflammatory environment to lipid metabolism and neuroendocrine response.

**Supplementary Information:**

The online version contains supplementary material available at 10.1186/s12944-024-02067-3.

## Background

Nowadays, chronic psychological stress has reached endemic levels across Western countries, contributing to a variety of diseases, like cardiovascular [1], neurodegenerative diseases [2], or endocrine disorders, such as diabetes mellitus (DM) [3]. The activation of physiological stress is mediated by the hypothalamus-pituitary-adrenal (HPA) axis and the sympathetic nervous system (SNS), leading to the production and release of glucocorticoids into systemic circulation [4]. Glucocorticoids (i.e., corticosterone (CORT) in rodents and cortisol in humans), the end-products of the HPA axis, are considered as key players in an organism’s response to stress. It is known that stress hormones not only mediate the stress response, but also regulate metabolism, inflammatory response, and immune function [5]. Since essential fatty acid (FA), omega (ω) 3, and ω6 polyunsaturated fatty acids (PUFAs) provide a foundation for the normal development and functioning of the brain and central nervous system (CNS), the development of neuropsychiatric disorders is associated with disturbances in FA intake and phospholipid metabolism [6–8]. ω3 PUFAs, mainly docosahexaenoic acid (C 22:6ω3) and eicosapentaenoic acid (C 20:5ω3), have strong anti-inflammatory, inflammation-resolving, anti-apoptotic, and anti-oxidative effects. Moreover, ω3 PUFAs antagonize the pro-inflammatory effects of ω6 PUFAs, as they are the precursors of pro-inflammatory mediators [9].

Disrupted lipid metabolism is also observed in DM, both type 1 and type 2, whose global incidence and prevalence rise dramatically each year [10, 11]. It has been shown that the cellular membranes of diabetics are rich in rigidity-promoting lipids, cholesterol, sphingomyelin, and saturated FAs (SFAs), causing the reduction of membrane fluidity and impaired insulin receptor signaling [12]. Defects in phospholipid membrane composition are also responsible for inflammation processes, resulting in impaired insulin secretion and signaling [12]. Moreover, pro-inflammatory cytokines can indirectly provoke oxidative stress (OS) by activating macrophages, which are known to play a key function in removing the pathogen by generating reactive oxygen species (ROS). As a result, an excess of ROS propagates nonenzymatic lipid peroxidation chain reactions that attack biomembranes [13] and directly damage phospholipids, resulting in membrane phospholipid oxidation [14].

The increased OS and endothelial activation are related to glucose excursions and directly tied to platelet hyperactivation [15], causing the generation of platelet-derived, highly instable thromboxane A2 (TxA2). The production of TxA2 is a sequential process that begins with arachidonic acid (C 20:4ω6), which is an ω6 PUFA present in the phospholipid membrane. TxA2 is considered as a potent vasoconstrictor of smooth muscles. It promotes platelet activation and aggregation shortly before being hydrolyzed into 11-dehydro-thromboxane B2 (11DHTXB2). As a stable metabolite, 11DHTXB2 is a reliable biomarker for platelet activity, which is highly associated with a oxidative/inflammatory environment significant for both DM and chronic psychological stress development and progression [16]. Therefore, this paper aims to determine and evaluate the impact of chronic psychological stress on the composition of the platelet phospholipid membrane and platelet activation in rats with type 1 diabetes mellitus (T1DM). This study provides insights into the deleterious effects of stress on the physical body, as chronic or prolonged exposure to psychological stress is generally associated with negative health outcomes. Therefore, mitigating the impact of stress is crucial for maintaining overall well-being.

## Methods

### Subjects of the study

This experimental study involved 35 mature (i.e., 7-month-old 19 subjects and 5-month-old 16 subjects) healthy female Wistar rats (weighing 253 g ± 13.1 g). Animals were randomly housed in cages of 3 rats each under standard conditions (i.e., room temperature 24 °C ± 1 °C, light/dark cycle: 12/12 h (light on at 8:00 and light off at 20:00), relative humidity: 55% ± 5%). The animals had free access to food and water during the experiment. Cages and bedding were replaced weekly. The studied animals were sourced from The Centre for Innovative Medicine, Vilnius, Lithuania.

### Study design

For the first 2 weeks, the animals (*n* = 35) were subjected to an adaptation period – rats were provided with standard laboratory rodent feed and water ad libitum without causing any stress. After adaptation, the animals were randomly divided into four groups, which were socially housed of three animals per cage and treated in different experimental conditions for 28 days. The rats were weighed on the 1st, 14th, and 28th experimental days, respectively.

The first group of rats (*n* = 9) was a control group that received water and standard feed ad libitum. No intervention was applied to the rodents during the experimental period.

Rats from the second group (*n* = 10) were subjected to chronic psychological stress. Restraint stress was produced daily for two hours by placing the animal in a small Plexiglas restraint cage with sufficient ventilation holes at both ends. No access to food and water was allowed during the procedure [17].

In the third group, rats (*n* = 8) were used to generate a streptozotocin-induced T1DM state. A single dose of 65 mg/kg of streptozotocin (MilliporeSigma, USA) treatment was used to establish diabetes using the procedure described in Furman’s published protocol [18] with minor corrections.

The rats in the fourth group (*n* = 8) underwent a single dose of 65 mg/kg of streptozotocin treatment to induce T1DM by Furman’s protocol and were subjected to stress of restraint in small Plexiglas restraint cages.

### Blood glucose monitoring

On the 1st and 28th experimental days, during the first three hours of the light cycle (i.e., to minimize diurnal variation effects), blood glucose levels were determined in all animals using the flavin-dependent glucose dehydrogenase method. Measurements were taken with two CONTOUR®PLUS ONE handheld glucometers (Ascensia Diabetes Care, Basel, Switzerland) using glucose test strips (Lot No. DP0MQHH07B). The utilization of two glucometers ensured accurate and validated blood glucose measurements while reducing the risk of device-related discrepancies. Whole blood was collected by pricking the lateral tail vein with a sterile needle, without the application of local analgesia or anesthesia. The glucometers have a measurement range of 0.6–32.0 mmol/L of glucose in whole blood and an accuracy of ± 0.5 mmol/L or ± 8.5%, as stated by the manufacturer. Notably, if the glucose level recorded exceeds 32.0 mmol/L, the device will display “HI” instead of providing a numerical result. Thus, such values were recorded as 32.0 mmol/L.

Blood glucose monitoring results revealed a statistically significant difference in glucose levels in T1DM and T1DM + Stress groups when comparing the 1st and 28th experimental days (T1DM group: 6.4 (1.95) mmol/L vs. 32.0 (1.13) mmol/L, *p* = 0.016; T1DM + Stress group: 6.8 (0.95) mmol/L vs. 32.0 (0.00) mmol/L). The results from the control and stress groups were not significant (control group: 6.3 (0.53) mmol/L vs. 6.9 (1.40) mmol/L, *p* = 0.322; stress group: 6.4 (0.93) mmol/L vs. 6.3 (0.45) mmol/L, *P* = 0.878). Therefore, experimental animals from T1DM and T1DM + Stress groups were considered diabetic.

### Blood sample collection

On the 29th day of the experiment, blood samples were collected from all rats (*n* = 35) between 10:00 and 12:00 in the morning (i.e., to avoid the peak time of CORT secretion). The collection was done through a cardiac puncture using a 20 G needle. Before the procedure, all rats were anesthetized using a mix of 80% CO2 and 20% O2 [19]. After blood collection, a cervical dislocation was performed to ensure a humane euthanasia of the animal.

To determine CORT and 11DHTXB2 concentrations in the blood serum, samples were collected in a 3.5 ml vacutainer tube with an inert gel barrier and clot activator. To analyze the composition of platelet membrane FAs, a 3 ml vacutainer tube with sodium heparin was used to collect the blood samples.

### Hair sample collection

Hair samples (i.e., approximately 150 mg per sample) were collected on days 1 and 29 from all experimental animals. On the 1st day, the rats were shaved to collect their hair samples from one side of each animal using an electric razor without local analgesia or anesthesia. The shaved area extended from the dorsal to the ventral midline and from neck to tail base. On day 29, the rats were shaved again to collect hair samples from the previously shaved sides of each animal after anesthesia and blood collection. Hair samples were wrapped in aluminum foil and stored in 10 mL polypropylene tubes at 4 °C until CORT extraction and analysis.

### Blood sample preparation

After the whole blood was drawn in vacutainer tubes for serum analysis, the samples were allowed to clot for 15–30 min at room temperature. The clot was removed by centrifuging at 1500 x g for 10 min in a refrigerated centrifuge. The serum samples were apportioned into 0.5 ml aliquots and stored at − 80 °C.

To obtain platelets, a vacutainer tube with an anticoagulant was subjected to centrifugation at 3000 x g for 10 min. Subsequently, three-quarters of the plasma were carefully removed without disturbing the cell and buffy coat layer. The remaining portion, rich in platelets (i.e., one-quarter), was separated, mixed with freezing media (provided by Biological Industries, Israel) at a ratio of 2:1, and frozen at a temperature of − 80 °C.

The flow cytometry method and anti-CD31, anti-CD42a antibodies were used to verify the platelet-rich fraction. The result showed that more than 90% of captured cells were of platelets’ origin.

### Hair sample preparation

Hair samples were prepared according to the slightly modified protocols published in previous studies [20, 21]. Samples were washed with 5 ml of HPLC-grade 2-isopropanol (Sigma-Aldrich, Germany) for 3 min, followed by the removal of the supernatant. Further, samples were dried in a protected hood at least 24 h at room temperature. Samples consisting of 20 mg of dried hair were weighed and transferred to 2 mL polypropylene tubes. Then, 1.5 mL of HPLC-grade methanol (Sigma-Aldrich, Germany) was added, and the samples were incubated for 24 h at room temperature on slow rotation. The samples were centrifuged at 10,000 g for 4 min at 4 °C, and the clear supernatant was transferred into the 2 mL polypropylene tubes. A stream of nitrogen gas was used for the evaporation of methanol and to dry the samples. The dry residue was resuspended in 0.2 mL of assay diluent provided in the CORT enzyme immunoassay kit.

#### Determination of blood serum and hair corticosterone

CORT concentration in serum and hair samples was measured using a commercially available enzyme immunoassay kit (CORT competitive ELISA kit, Thermo Fisher Scientific Inc., Carlsbad, CA; Catalog No. EIACORT). The analytical sensitivity of the assay was 18.6 pg/mL, the intra-assay CV ranged from 3.1 to 6.5% for the 4 quality controls conducted by the manufacturer. Cross-reactivity was as follows: desoxycorticosterone 12.30%, tetrahydrocorticosterone 12.30%, aldosterone 0.62%, cortisol 0.38%, progesterone 0.24%, dexamethasone 0.12%, < 0.1% corticosterone-21-hemisuccinate, < 0.08% cortisone and estradiol. CORT levels in serum samples are reported in ng/mL, and in hair samples – pg/mg.

### Determination of platelet membrane fatty acids

After allowing the samples to thaw at room temperature, the Folch method [22] was used to extract the lipids from the platelet membrane. Subsequently, thin-layer chromatography was performed (Sil G-25 UV 254) to separate the platelet membrane phospholipids [23]. Following transesterification, gas chromatography-mass spectrometry was conducted using a GCMS-QP2010 Ultra (Shimadzu, Japan) analyzer to identify the methyl esters of FAs. The obtained data were then collected and processed using LabSolutions (Shimadzu, Japan) software version 4.52.

The content of each FA in the total FA amount (100%) was determined by calculating the percentage of SFAs (i.e., C 14:0, C 16:0, C 18:0), monounsaturated FAs (MUFAs) (i.e., C 16:1ω7, C 18:1ω9, C 18:1ω7, C 20:1ω9), PUFAs (i.e., C 18:2ω6, C 18:3ω3, C 20:4ω6, C 20:5ω3, C 22:5ω3, C 22:6ω3), and the percentage of PUFAs ω3 and ω6.

### Determination of blood serum 11-dehydro thromboxane B2

A quantitative determination of rat 11DHTXB2 in blood serum was performed using a commercially available enzyme immunoassay kit (11DHTXB2 competitive ELISA kit, MyBioSource Inc., San Diego, CA; Catalog No. MBS289362). The sensitivity of the assay was 1.0 pg/mL. CV in the same lot and a different lot was less than 10% according to the manufacturer. No significant cross-reactivity or interference between 11DHTXB2 and an analogue was observed. Levels of blood serum 11DHTXB2 are presented in pg/mL.

### Statistical analysis

The statistical analysis was carried out using the R Studio and R Commander software packages. MS Excel 2019 was used for visual representation. As the total number of subjects was 35, nonparametric tests were performed. For more precise correlations between the composition of platelet membrane FAs, the concentrations of hair and blood serum CORT, and 11DHTXB2, nonparametric Spearman’s correlation coefficient was applied.

This study presents data in the median (Mdn), minimum, and maximum values, along with the interquartile range (IQR). The Kruskal-Wallis test was used to determine differences in the median values of distinct biomarkers between the four experimental groups. The Mann-Whitney-Wilcoxon test was used as a post hoc test to determine the differences between each of the groups. Statistical significance was determined by a *P*-value below 0.05.

## Results

### Body weight

**Fig. 1:**
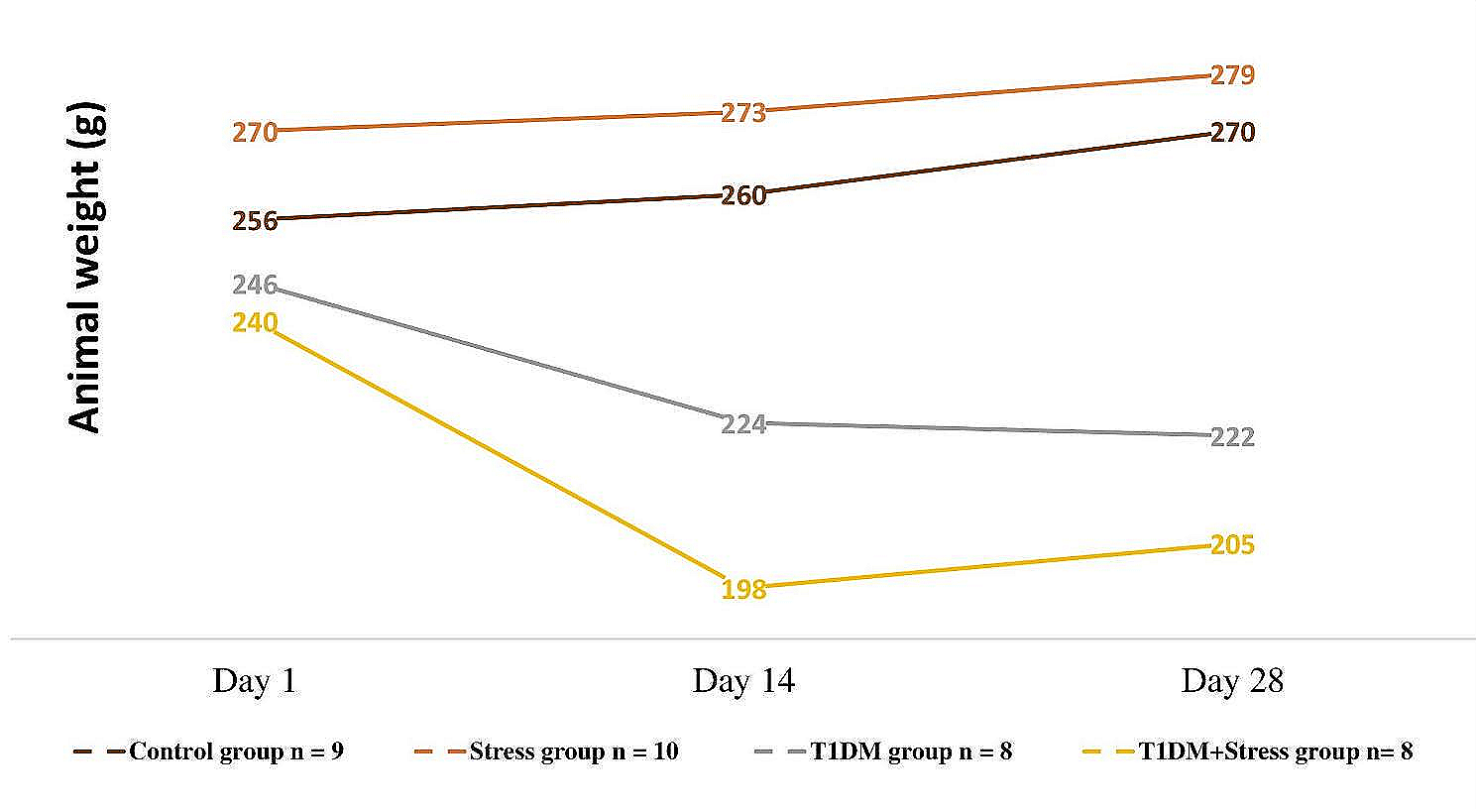
A comparison of animal body mass dynamics across different experimental groups. The data are presented in median

Animal body weight (Fig. 1) increased by 2.2% in the stress group comparing the 14th and 28th experimental days (Mdn = 273 (IQR = 13.5) g vs. Mdn = 279 (IQR = 16.5) g, *P* = 0.009) and by a total 3.3% increase comparing the 1st and 28th experimental days (Mdn = 270 (IQR = 17.8) g vs. Mdn = 279 (IQR = 16.5) g *P* = 0.040). Rats from T1DM and T1DM + Stress groups showed a statistically significant decrease of body weight. A total 9.8% weight reduction was observed in the T1DM group when comparing the 1st and 28th experimental days (Mdn = 246 (IQR = 9.5) g vs. Mdn = 222 (IQR = 16.0) g, *P* = 0.016); a 8.9% of total weight reduction was reached during the first 14 days (Mdn = 246 (IQR = 9.5) g vs. Mdn = 224 (IQR = 17.0) g, *P* = 0.031). By comparing rats’ body mass in the T1DM + Stress group between the 1st and 14th days, a clear 17.5% weight loss (Mdn = 240 (IQR = 10.0) g vs. Mdn = 198 (IQR = 29.0) g, *P* = 0.014) and a total 14.5% mass reduction was observed between 1st and 28th experimental days (Mdn = 240 (IQR = 10.0) g vs. Mdn = 205 (IQR = 33.0) g, *P* = 0.014). In the same group, there was a slight 3.0% increase of body weight during the last fourteen days (Mdn = 198 (IQR = 29.0) g vs. Mdn = 205 (IQR = 33.0) g, *P* = 0.269). Rats from the control group also had a total 5.5% increase of body mass (Mdn = 256 (IQR = 7.0) g vs. Mdn = 270 (IQR = 11.8) g). The results, however, were not statistically significant (*P* = 0.253).

### Blood serum and hair corticosterone

**Fig. 2:**
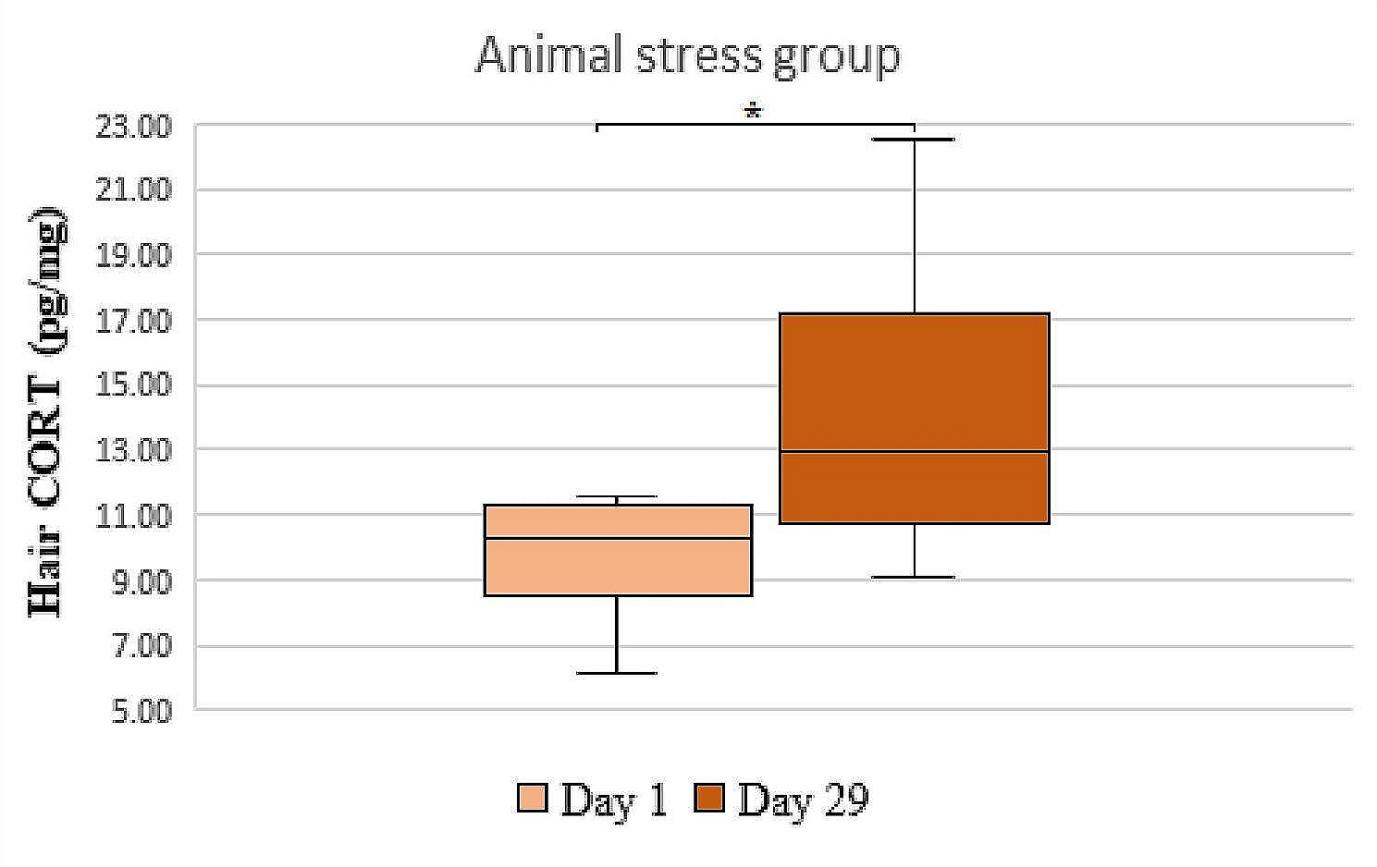
A comparison of hair corticosterone concentrations in the animal stress group. * *P* = 0.027, *n* = 10

Blood serum CORT concentration (Table 1) was significantly lower in T1DM and in T1DM + Stress groups as compared to the stress group (*P* = 0.031, *P* = 0.008). Rats with T1DM also had a lower concentration of CORT in their blood serum than those in the control group (*P* = 0.031). However, the concentration of CORT in hair samples was statistically significantly higher in the stress group when comparing the 1st and 29th experimental days (*P* = 0.027) (Fig. 2); likewise, the concentration of CORT in their blood serum was statistically significantly higher when compared to other experimental groups (Table 1). However, the results of hair CORT in T1DM and in T1DM + Stress groups showed no significant difference.

**Table 1:** A comparison of blood serum corticosterone concentrations among different experimental groups

Blood serum (Units)	Median, minimum, maximum, interquartile range	Control group (*n* = 9)^1^	Stress group (*n* = 10)^2^	T1DM group (*n* = 8)^3^	T1DM + Stress group (*n* = 8)^4^	P-values*
Corticosterone (ng/mL)	Median	326.10	549.30	149.45	126.65	1 vs. 2 = 0.148 **1** ***v*** **s 3 = 0.031** 1 vs. 4 = 0.219 **2** vs. **3 = 0.031** **2** vs. **4 = 0.008** 3 vs. 4 = 1.00
	Minimum	16.02	28.08	51.26	56.28	
	Maximum	632.90	899.40	515.20	640.90	
	IQR	95.25	307.91	174.57	43.81	

T1DM – type 1 diabetes mellitus, IQR – interquartile range. * Mann-Whitney-Wilcoxon test

### Platelet membrane FA composition

**Fig. 3:**
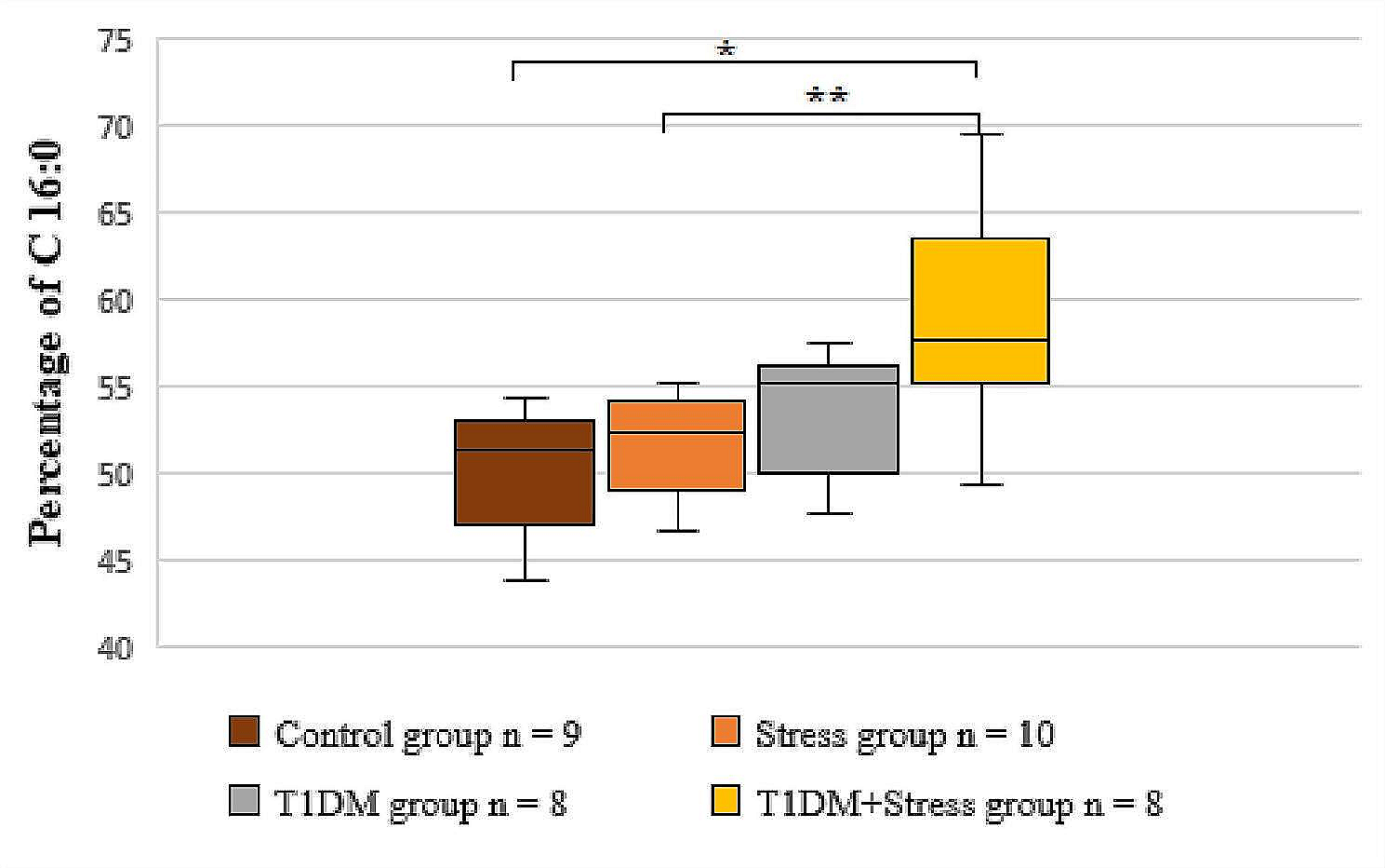
A comparison of C 16:0 percentages in the platelet membrane across different experimental groups. * *P* = 0.016, ** *P* = 0.055

The composition of FAs in platelet phospholipid membranes is listed in Table 2. An analysis of SFAs showed that the level of C 14:0 was lower in the stress and in T1DM + Stress groups as compared to control (*P* = 0.023, *P* = 0.047), but C 16:0 was observed to be higher in the T1DM + Stress group (*P* = 0.016) as compared to the control group. Rats from the same experimental group (i.e., T1DM + Stress group) also had a higher level of C 16:0 as compared to the stress group (*P* = 0.055) (Fig. 3), but their level of C 18:0 was lower (*P* = 0.008). Moreover, C 18:0 of platelet phospholipid membrane decreased in the T1DM group as compared to stress group rats (*P* = 0.031).

**Table 2:** A comparison of the composition of FAs in rats’ platelet membranes across different experimental groups

Platelet FAs (provided by percentage of total amount)	Median, minimum, maximum, interquartile range	Control group (*n* = 9)^1^	Stress group (*n* = 10)^2^	T1DM group (*n* = 8)^3^	T1DM + Stress group (*n* = 8)^4^	*P*-values
C 14:0^*^	Median	4.77	3.47	2.60	1.87	**1** vs. **2 = 0.023**^**$**^ 1 vs. 3 = 0.219 **1** vs. **4 = 0.047** 2 vs. 3 = 1.000 2 vs. 4 = 0.383 3 vs. 4 = 0.563
	Minimum	2.95	1.98	1.64	1.40	
	Maximum	6.56	4.68	6.45	5.84	
	IQR	0.88	0.81	1.08	1.33	
C 16:0	Median	51.40	52.43	55.15	57.66	1 vs. 2 = 0.742 ^**$**^ 1 vs. 3 = 0.156 **1** vs. **4 = 0.016** 2 vs. 3 = 0.563 **2** vs. **4 = 0.055** 3 vs. 4 = 0.313
	Minimum	43.83	46.66	47.68	49.30	
	Maximum	54.38	55.2	57.58	69.44	
	IQR	4.63	4.29	2.96	6.53	
C 18:0	Median	38.41	39.60	37.35	34.50	1 vs. 2 = 0.195 ^**$**^ 1 vs. 3 = 0.313 1 vs. 4 = 0.297 **2** vs. **3 = 0.031** **2** vs. **4 = 0.008** 3 vs. 4 = 0.844
	Minimum	31.39	33.64	27.82	20.06	
	Maximum	42.85	43.62	46.54	38.43	
	IQR	6.19	2.95	4.54	9.46	
C 16:1ω^**^7	Median	0.69	0.30	0.23	0.52	0.199^**#**^
	Minimum	0.39	0.11	0.14	0.03	
	Maximum	3.07	1.26	2.19	1.27	
	IQR	0.76	0.50	0.48	0.67	
C 18:1ω7	Median	0.51	0.34	0.20	0.39	1 vs. 2 = 0.461 ^**$**^ 1 vs. 3 = 0.313 **1** vs. **4 = 0.047** 2 vs. 3 = 0.313 2 vs. 4 = 0.945 3 vs. 4 = 0.916
	Minimum	0.10	0.04	0.06	0.21	
	Maximum	1.78	1.54	1.35	0.85	
	IQR	0.83	0.78	0.33	0.31	
C 18:1ω9	Median	1.70	1.03	0.81	1.40	0.568^**#**^
	Minimum	0.43	0.10	0.19	0.31	
	Maximum	4.35	2.81	4.37	3.42	
	IQR	1.19	1.45	1.04	0.75	
C 20:1ω9	Median	0.31	0.26	0.24	0.17	1 vs. 2 = 0.148 ^**$**^ 1 vs. 3 = 0.156 **1** vs. **4 = 0.016** 2 vs. 3 = 0.438 **2** vs. **4 = 0.068** **3** vs. **4 = 0.036**
	Minimum	0.19	0.14	0.11	0.12	
	Maximum	0.59	0.41	0.42	0.21	
	IQR	0.16	0.17	0.07	0.04	
C 18:2ω6	Median	1.91	1.205	1.55	2.00	0.580^**#**^
	Minimum	0.33	0.43	0.65	0.72	
	Maximum	5.55	4.94	8.20	8.02	
	IQR	1.36	1.61	1.00	1.19	
C 18:3ω3	Median	0.59	0.32	0.28	0.25	1 vs. 2 = 0.148 ^**$**^ 1 vs. 3 = 0.400 **1** vs. **4 = 0.031** 2 vs. 3 = 0.313 2 vs. 4 = 0.483 3 vs. 4 = 0.688
	Minimum	0.43	0.17	0.13	0.11	
	Maximum	0.82	0.92	1.03	0.92	
	IQR	0.20	0.15	0.25	0.25	
C 20:4ω6	Median	0.49	0.33	0.28	0.19	0.285^**#**^
	Minimum	0.10	0.06	0.10	0.13	
	Maximum	1.94	1.39	0.6	1.08	
	IQR	0.42	0.54	0.20	0.26	
C 20:5ω3	Median	0.29	0.12	0.08	0.06	1 vs. 2 = 0.195 ^**$**^ **1** vs. **3 = 0.031** **1** vs. **4 = 0.016** 2 vs. 3 = 0.787 2 vs. 4 = 0.262 3 vs. 4 = 0.563
	Minimum	0.09	0.02	0.02	0.01	
	Maximum	0.40	0.38	0.21	0.25	
	IQR	0.09	0.09	0.06	0.04	
C 22:5ω3	Median	0.08	0.06	0.07	0.06	**1** ***v*** **s 2 = 0.079** ^**$**^ **1** vs. **3 = 0.093** 1 vs. 4 = 0.375 2 vs. 3 = 0.343 2 vs. 4 = 0.554 3 vs. 4 = 0.590
	Minimum	0.05	0.03	0.03	0.01	
	Maximum	0.14	0.09	0.15	0.14	
	IQR	0.06	0.04	0.06	0.06	
C 22:6ω3	Median	0.07	0.07	0.08	0.05	1 vs. 2 = 1.000 ^**$**^ 1 vs. 3 = 1.000 1 vs. 4 = 0.178 2 vs. 3 = 0.844 **2** vs. **4 = 0.016** 3 vs. 4 = 0.156
	Minimum	0.02	0.04	0.03	0.02	
	Maximum	0.12	0.13	0.16	0.07	
	IQR	0.05	0.05	0.07	0.02	
Σ^***^ SFAs	Median	92.80	95.77	96.29	94.32	0.428^**#**^
	Minimum	81.63	87.27	83.01	85.21	
	Maximum	96.89	98.63	97.3	97.71	
	IQR	3.79	5.57	2.90	3.10	
Σ MUFAs	Median	3.64	1.89	1.31	2.59	0.420^**#**^
	Minimum	1.41	0.41	0.93	0.73	
	Maximum	9.71	5.73	7.78	5.23	
	IQR	2.32	2.87	1.85	1.71	
Σ PUFAs	Median	3.57	2.32	2.48	3.03	0.440^**#**^
	Minimum	1.70	0.96	1.56	1.57	
	Maximum	8.66	7.01	9.24	9.56	
	IQR	1.48	2.71	1.02	1.68	
Σ ω3	Median	1.07	0.60	0.60	0.42	1 vs. 2 = 0.109 ^**$**^ 1 vs. 3 = 0.156 **1** vs. **4 = 0.031** 2 vs. 3 = 0.313 2 vs. 4 = 0.195 3 vs. 4 = 0.438
	Minimum	0.70	0.29	0.24	0.19	
	Maximum	1.41	1.45	1.31	1.31	
	IQR	0.17	0.24	0.25	0.32	
Σ ω6	Median	2.45	1.53	1.86	2.18	0.597^**#**^
	Minimum	0.43	0.49	0.78	0.85	
	Maximum	7.49	6.33	8.8	9.1	
	IQR	1.90	2.19	1.15	1.48	

SFAs – saturated fatty acids, MUFAs – monounsaturated fatty acids, PUFAs – polyunsaturated fatty acids, T1DM – type 1 diabetes mellitus, FAs – fatty acids, IQR – interquartile range, * – number of carbon atoms and double bonds, ^**^ – position of double bond between carbon atoms in the molecule, ^***^ – total sum, ^$^Mann-Whitney-Wilcoxon test, ^#^Kruskal-Wallis test

**Fig. 4:**
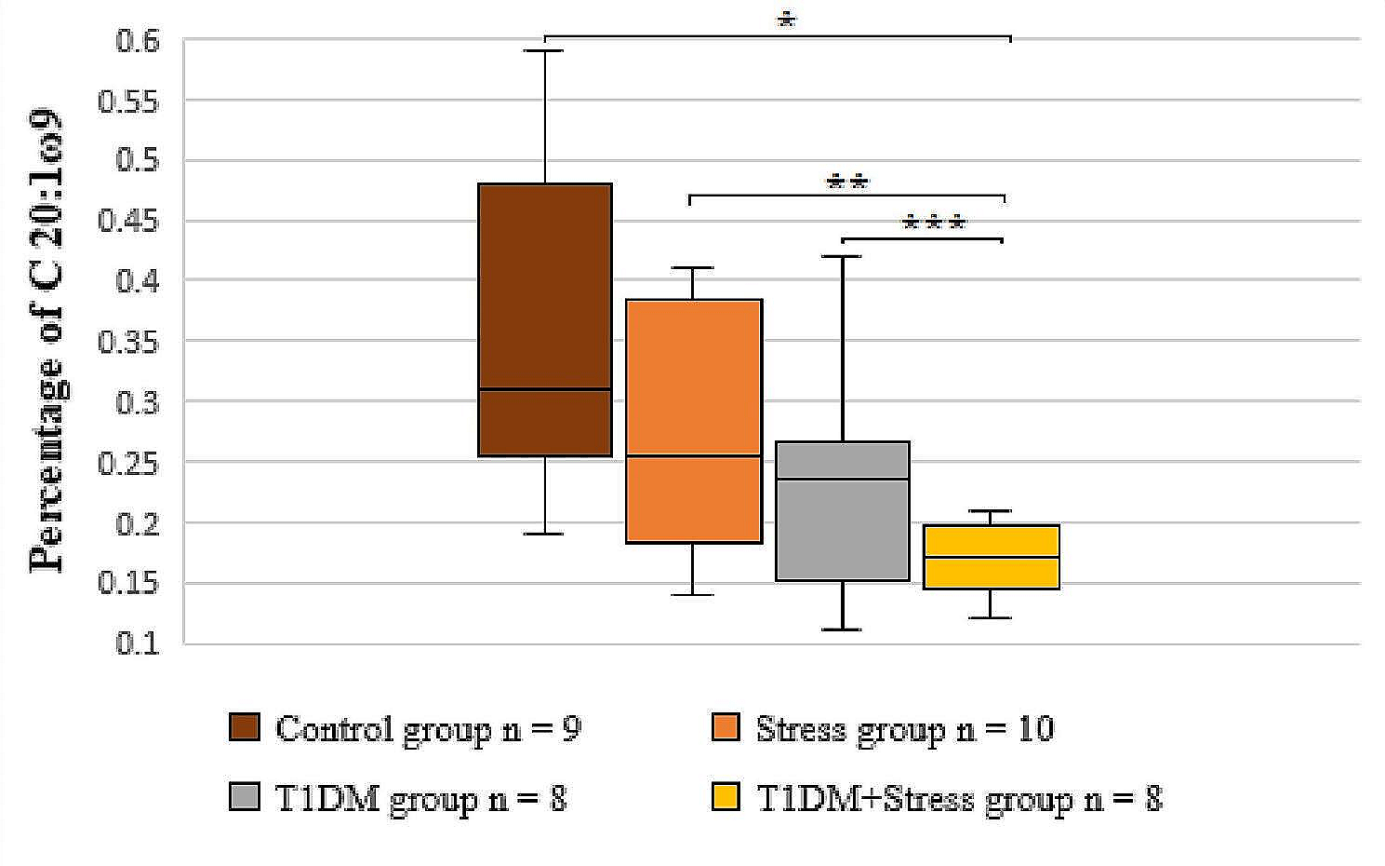
A comparison of C 20:1ω9 percentages in the platelet membrane across different experimental groups. * *P* = 0.016, ** *P* = 0.068, *** *P* = 0.036

By analyzing the results of MUFAs in platelet phospholipid membranes, less C 18:1ω7 and C 20:1ω9 were observed in the T1DM + Stress group than in the control rat group (*P* = 0.047; *P* = 0.016). Furthermore, the tendency of a lower level of C 20:1ω9 was also observed in the T1DM + Stress group, when comparing to the stress group (*P* = 0.068) and the T1DM group separately (*P* = 0.036) (Fig. 4).

**Fig. 5:**
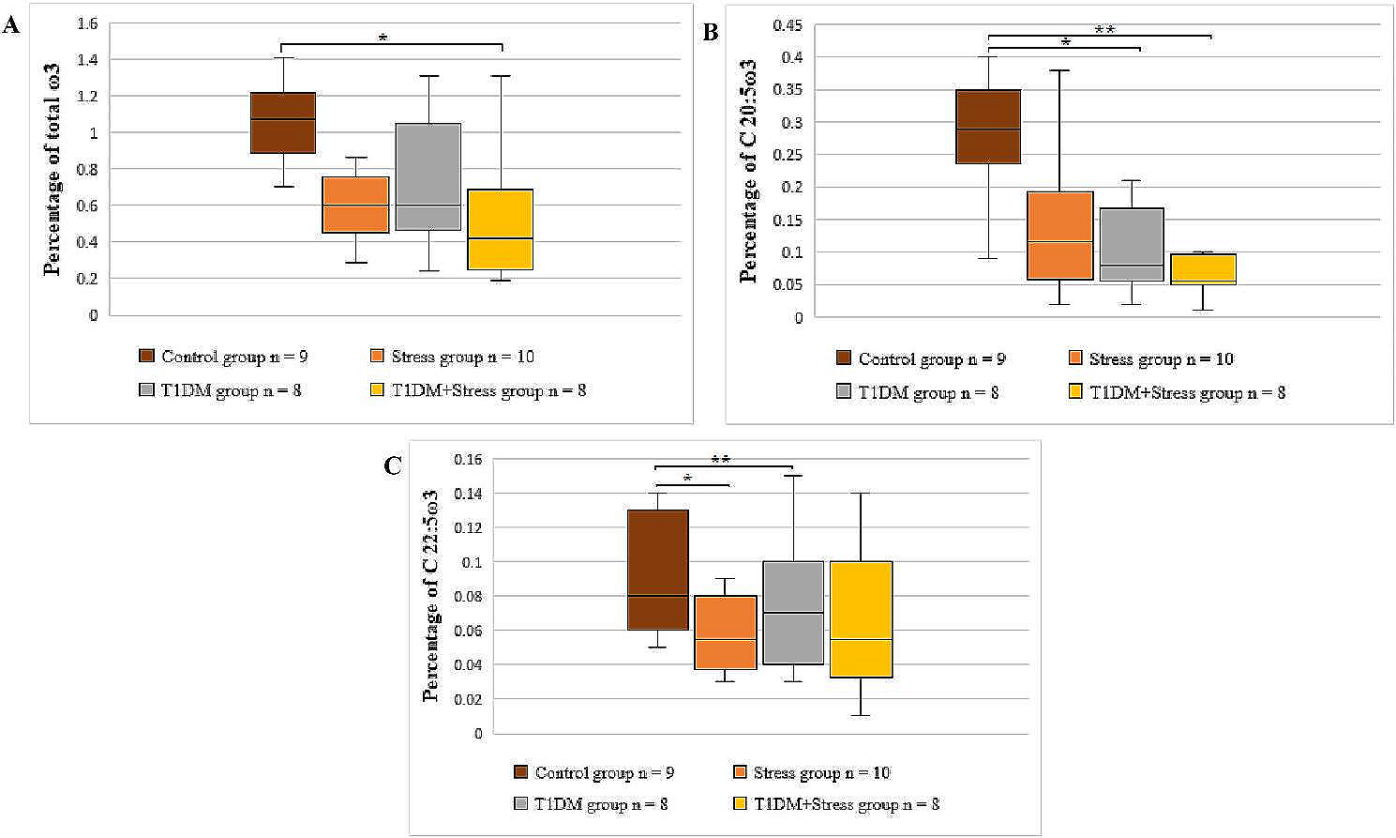
A comparison of ω3 PUFAs in platelet membrane across different experimental groups. (**A**) shows the percentage of total ω3, * *P* = 0.0313; (**B**) shows the percentage of C 20:5ω3, * *P* = 0.031, ** *P* = 0.016; (**C**) shows the percentage of C 22:5ω3, * *P* = 0.079, ** *P* = 0.093

The total sum of ω3 PUFAs was lower in all intervention groups than in control animals. Moreover, the lowest level of total ω3 PUFAs was in T1DM + Stress group compared to control (*P* = 0.031) (Fig. 5, panel A). The percentages of C 18:3ω3 and C 20:5ω3 PUFAs also were statistically significantly lower in platelet phospholipid membranes of the T1DM + Stress group as compared to control rats (*P* = 0.031; *P* = 0.016). The results of C 20:5ω3 and C 22:5ω3 (Fig. 5, panel B and C) showed a decreasing level tendency in rats with T1DM compared to the control group (*P* = 0.031, *P* = 0.093). A similar tendency was observed for C 22:5ω3 in the stress group in comparison with control (*P* = 0.079). However, experimental rats from the stress group had a higher percentage of C 22:6ω3 in their platelet phospholipid membranes than rats from the T1DM + Stress group (*P* = 0.016).

### Blood serum 11-dehydro thromboxane B2

**Fig. 6:**
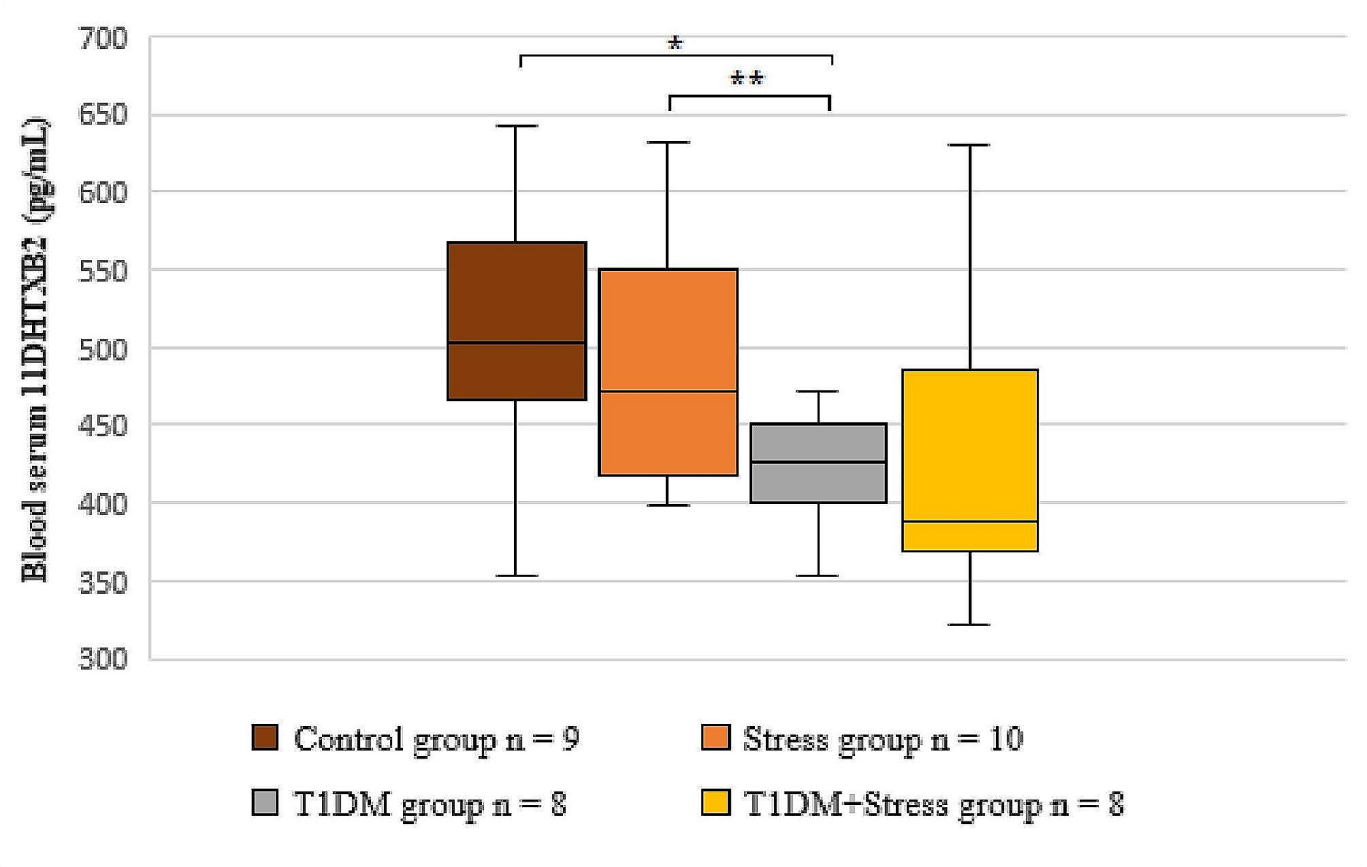
A comparison of blood serum 11-dehydro thromboxane B2 concentrations across different experimental groups. * *P* = 0.094; ** *P* = 0.063

When examining the results of the 11DHTXB2, a consistent trend of decreasing concentrations was observed among all the experimental rat groups as compared to the control group, as depicted in Fig. 6. Rats with T1DM exhibited reduced levels of 11DHTXB2 in their blood serum when compared to both the control and stress groups individually (*P* = 0.094; *P* = 0.063).

### Correlation results

In stress group, blood serum CORT showed a correlation with C 18:3ω3 (*r* = -0.6280605, *P* = 0.052) and 11DHTXB2 (*r* = -0.6848485, *P* = 0.035). A moderate direct correlation between C 18:0 and 11DHTXB2 (*r* = 0.6363636, *P* = 0.054) in the stress group was observed as well (*r* = 0.6707442, *P* = 0.034). There was also a moderate direct correlation between C 18:3ω3 and C 20:5ω3 in the T1DM group (*r* = 0.682647, *P* = 0.062), and between C 18:3ω3 and C 22:5ω3 (*r* = 0.7831325, *P* = 0.022) in the T1DM + Stress group.

## Discussion

According to our study results, rats in the restraint stress group (i.e., a simulation for chronic psychological stress such as occupation-related stress in humans [17]) had the highest level of hair and blood serum CORT as compared to other experimental groups. Moreover, rats in the stress group had a significant weight gain compared with the other groups. Such results were expected, as it is widely known that stress has a major effect on metabolic activity and stimulates the release of various hormones. Increasing the concentration of CORT stimulates insulin secretion in nondiabetic rats and humans. The high level of glucocorticoids, accompanied by a high concentration of insulin, exerts a strong anabolic effect on fat, particularly abdominal fat, resulting in weight gain [24, 25]. However, rats in the T1DM and in T1DM + Stress groups clearly demonstrated lower concentrations of blood serum CORT followed by a significant decrease of body mass. These results could likely be attributed to various factors, including habituation, fatigue, or a combination of both. According to the scientific data of other authors, long-term hypokinesia or repeated stress may lead to an absence of the adrenal response; thus, rats may elevate their CORT levels mostly to new stimuli. It must also be noted that generally, physical stress induces a greater hormonal response than psychological stress. Secondly, when the stimuli are more severe and weight loss occurs (mesenteric and other fat stores decrease), due to induced metabolic disorder (i.e., reduction of insulin secretion) and greater sensitivity, rodents may develop an exhaustion (i.e., the state when an animal can no longer compensate and their condition becomes life-threatening) [26, 27]. Additionally, CORT, the primary glucocorticoid produced in the adrenal cortex of rats, may decrease due to the loss of body fat, which serves as the initial substrate for the synthesis of steroid hormones [28].

By analyzing the platelet membrane phospholipid FA results acquired in the present study an increase of SFA (i.e., C 16:0, C 18:0) was observed in rats experiencing chronic psychological stress as compared to the control group. Similar results were published in Bernardi’s et al. paper [29]. Its authors assessed an early stressful event, such as the maternal separation of Wistar rat litters, and interaction with the nutritional availability of ω3 PUFAs during the life course. Their data showed that maternal-separated rats with an adequate diet of ω3 PUFAs had a higher level of SFAs in their peripheral blood than non-handled rats with the same diet. Moreover, maternal separated rats had an increased abdominal fat deposition resulting in body weight gain compared to the non-handled ones. A likewise tendency was observed in our study. Hennebelle et al. [30] also noticed similar results in their research, analyzing FAs in the plasma of rats fed with a control diet and having repeated restraint stress as compared to rats with the same diet and no stress. These rats had a plasma CORT increase, as the one observed in our study. Scientific data of other authors note that elevated plasma-free FAs correlate with certain psychological alterations. SFAs, especially C 16:0, induce anxiety-like behavior while increasing amygdala-based serotonin metabolism. Furthermore, C 16:0 has the potential to be lipotoxic to cells by causing the accumulation of ROS, which can result in endoplasmic reticulum (ER) stress and ultimately lead to cell apoptosis in both humans and model organisms, as described by Moon et al. [31]. ER, consequently, can result in cellular dysfunction and diseases such as neurodegeneration and DM [32].

Our experimental animals with T1DM and T1DM + Stress showed a clear decrease of C 14:0, C 18:0 and an increase of C 16:0 accompanied by a significant body weight loss. The parallel results were observed in a study by Shen et al. [33], which analyzed the composition of plasma FAs in healthy rats and in rats with T1DM induced by streptozotocin. Moreover, patients with T1DM and bad glycemic control also had similar SFA results in their plasma as compared to healthy controls in a study by Sobczak et al. [34]. Elevated levels of the total sum of SFAs in the T1DM groups could be due to SFA C 16:0 serving as an energy source or a building block for lipid metabolism, as well as its pathogenic roles: working as a signaling molecule that regulates the progression and development of many diseases at the molecular level. Recent scientific studies demonstrate that a high level of plasma C 16:0 increases the cellular uptake of C 16:0, leading to insulin signaling inhibition and the development of insulin resistance. Furthermore, in pancreatic islets, C 16:0 inhibits glucose-induced insulin secretion by impairing exocytosis evoked by action potential-like depolarization. SFA C 16:0 also increases the production of interleukin (IL) 1β that not only promotes the development of insulin resistance in tumor necrosis factor (TNF)-dependent and TNF-independent pathways, but also activates autophagy by inducing ER stress and causing metabolic dysregulation [35].

Our study showed a lower percentage of MUFAs in all intervention groups as compared to the control group. The data of decreased MUFAs in plasma were also noticed in rats with repeated restraint stress [30]. Moreover, Juncker et al. [36] studied the FA composition of human milk in women experiencing postpartum stress. They noticed that women who experienced higher levels of stress had a significantly lower levels of MUFAs in mature milk than women in a control group. There is increasing evidence linking MUFAs to an anti-inflammatory effect, as they may lower levels of circulating mononuclear cells (i.e., monocytic cells are involved in the inflammatory response), increase circulating anti-inflammatory markers (IL-4 and IL-10), and lower proinflammatory markers (IL-6, monocyte chemoattractant protein-1, IL-1 and TNF-α). In addition, MUFAs can generate a more favorable plasma lipid profile and increase cognitive function [37]. Thus, we assume that our experimental animals with declined MUFA levels might have underwent an inflammatory status leading to increased MUFAs oxidation, as they were more sensitive to peroxidation than SFAs.

The same explanation could be applied to T1DM and T1DM + Stress rats, as low-grade inflammation has also been associated with DM. Furthermore, it is known that MUFAs are related to anti-diabetic effects by improving the insulin receptor substrate 1/phosphoinositide 3-kinase insulin signaling pathway, activating the adenosine monophosphate-activated protein kinase, and translocating glucose transporter 4. The anti-hyperglycemic effect is also observed, as MUFAs may enhance insulin sensitivity and glucose uptake, and they may inhibit hepatic gluconeogenesis or other relevant insulinotropic actions [38]. Therefore, we support the hypothesis that the abovementioned processes could have been altered in our experimental subjects due to a lack of MUFAs, which was also observed in other DM animal studies [39, 40].

PUFAs, especially ω3 PUFAs, have a wide range of effects on mental health at the molecular and cellular levels, as they optimize membrane fluidity and lipid bilayer elasticity. ω3 PUFAs improve ion channel function and the binding of neurotransmitters and their receptors in the membrane, stimulate the expansion of cell membranes at the nerve growth cones, and regulate the activity of signal molecules, gene expression, and epigenetic modifications. Moreover, ω3 PUFAs may antagonize inflammation and modulate the immune response, affect mitochondrial function and ROS homeostasis, cell proliferation, viability and cell repair, or apoptosis [9]. However, epidemiological and animal studies show a negative correlation between the status of ω3 PUFAs and stress-associated disorders, such as anxiety and depression. Laurego et al. [41] demonstrated in their study that chronically stressed titi monkeys (*C. cupreus*) had a lower plasma ω3 FA status than the control group. Larrieu et al. [42] also noticed that a deficiency of dietary ω3 PUFAs induces the chronic stress phenotype in mice. Moreover, they measured HPA axis activity and found that an ω3 deficiency induced a significant increase in plasma CORT levels in undefeated ω3-deficient mice as compared to undefeated control diet mice. Similar results were obtained in our study, where chronically restrained rats had a lower level of ω3 PUFAs, a higher concentration of hair and blood serum CORT, and a clearly negative correlation between C 18:3ω3 and blood serum CORT. Moreover, the results showed that the lower C 18:3ω3 (i.e., the main substrate of ω3 long-chain PUFA biosynthesis) percentage gets, the lower level of C 20:5ω3 is detected. This clearly indicates that the organism may develop a disrupted lipid metabolism due to overuse of chronic stress.

The levels of total ω3 PUFAs and certain ω3 FAs (i.e., C 18:3ω3, C 20:5ω3, C 22:5ω3) also decreased in T1DM and T1DM + Stress rats as compared to the control group in the present study. Moreover, the decline in percentage of C 18:3ω3 resulted in decreased levels of C 20:5ω3 and C 22:5ω3 in the T1DM and T1DM + Stress groups, respectively. Yao et al. [43] demonstrated similar results: DM rats had a lower level of C 18:3ω3, C 20:5ω3, and C 22:5ω3 PUFAs in their liver samples than those of the control group. Krishna Mohan and Das [44] found a significantly lower level of plasma PUFAs in alloxan-induced T1DM compared to an untreated group. Studies show that free-radical generation is increased in diabetic animals and in patients with T1DM and type 2 DM. Moreover, pancreatic antioxidant enzymes, such as superoxide dismutase, glutathione peroxidase, and catalase, exhibit less activity, which leads to enhanced formation of free radicals in diabetes. Therefore, these processes might accelerate lipid peroxidation [44]. Moreover, it is known that FA desaturases (i.e., Δ5- and Δ6-desaturase) are the key enzymes for the biosynthesis of PUFAs. The absence of insulin in animal models and in T1DM patients results in a marked decrease in the biosynthesis of PUFAs, including C 20:4ω6, because of the lower transcription and lower activity of Δ5- and Δ6-desaturases [43]. Therefore, declining levels of ω3 PUFAs could be observed.

DM is associated with OS due to intracellular hyperglycemia, or an increased oxidation of FAs and superoxide, and low antioxidant capacity. Moreover, low-grade inflammatory stimuli also induce an increased lipid peroxidation with consequent platelet activation, resulting in thromboxane A2 (TxA2) and further OS [15]. Enzymatically produced TxA2 from C 20:4ω6 is hydrolyzed into a biologically inactive but more stable thromboxane B2 (TxB2) metabolite. TxB2 is further metabolized primarily into a 11DHTXB2 form [16]. 11DHTXB2 is a marker of platelet activity and inflammation, which increases in DM subjects, both humans and experimental animals [15, 45]. However, the findings of this study demonstrate the contrary: all experimental groups had lower levels of 11DHTXB2 as compared to control rats, although the stress group had the highest concentration among other affected groups. The explanation for this could probably be traced to an imbalance between the production and accumulation of ROS, a common factor in DM and chronic stress, leading to lipid peroxidation and a declined activity of the aforementioned desaturases. Therefore, decreasing levels of C 20:4ω6 in rat platelet phospholipid membranes, where C 20:4ω6 is the main precursor of 11DHTXB2 biosynthesis, might be observed. The decrease was especially evident in the present study, which recorded a negative correlation between blood serum CORT and 11DHTXB2 of the stress group.

### Study strengths and limitations

The main strength of this study was that the relationship between chronic psychological stress, alteration in platelet membrane FA, and increased platelet activation in T1DM was evident, although experimental subject groups were small. In addition, the model of chronic stress chosen for the experimental animals simulated occupation-related stress, which is considered one of the most common chronic stressors in humans.

There were also a few potential limitations in the present study. First, it measured a biomarker of inflammation, but there was no evaluation of oxidative stress and/or activation of antioxidant enzymes, even though both DM and chronic psychological stress undergo such conditions simultaneously. Second, we did not consider applying insulin and/or a reduction of chronic psychological stimuli, or even certain FAs, as treatment options in order to get the reverse outcome, though scientific data declare that most of the abnormalities revert to normal conditions by applying the aforementioned treatment.

## Conclusions

The findings of the present study indicate that chronic psychological stress leads to increased production of the primary stress hormone, CORT, and heightened platelet activation within the systemic circulation. Additionally, the percentage of SFAs, particularly C 16:00, was found to be elevated in the platelet phospholipid membrane both in the stress and T1DM groups. The levels of MUFAs (i.e., C 18:1ω7, C 20:1ω9) and PUFAs, especially ω 3 FAs (i.e., C 18:3ω3, C 20:5ω3, C 22:5ω3, C 22:6ω3), were lower in chronically stressed animals. This clearly indicates the disruptive impact of chronic stress and DM to lipid metabolism and the neuroendocrine response. These processes are highly related to the inflammatory and oxidative status in the animal model; they can induce a hypercoagulable state, leading to the development and progression of cardiovascular outcomes.

### Electronic supplementary material

Below is the link to the electronic supplementary material.

Supplementary Material 1

## Data Availability

No datasets were generated or analysed during the current study.

## Abbreviations

DM: Diabetes mellitus
HPA: Hypothalamus-pituitary-adrenal axis
SNS: Sympathetic nervous system
CORT: Corticosterone
FA: Fatty acid
Ω: Omega
PUFAs: Polyunsaturated fatty acids
CNS: Central nervous system
SFAs: Saturated fatty acids
ROS: Reactive oxygen species
11DHTXB2: 11-dehydro-thromboxane B2
T1DM: Type 1 diabetes mellitus
MUFAs: Monounsaturated fatty acids
ER: Endoplasmic reticulum
IL: Interleukin
TNF: Tumor necrosis factor
