# Supplementary material for: Impact of chronic psychological stress on platelet membrane fatty acid composition in a rat model of type 1 diabetes Mellitus
Source: Lipids Health Dis. 2024 Mar 8;23:69. doi: 10.1186/s12944-024-02067-3 (PMC10921692; doi:10.1186/s12944-024-02067-3)
Supplement: Supplementary file 1 — Supplementary Material 1 [file 12944_2024_2067_MOESM1_ESM.pdf]

Impact of Chronic Psychological Stress on Plate...

By: inga bikulciene

As of: Feb 2, 2024 6:32:44 AM
6,597 words - 128 matches - 66 sources

Similarity Index

19%

Mode: Similarity Report

paper text:

Impact of Chronic Psychological Stress on Platelet Membrane Fatty Acid Composition in Type 1 Diabetes Mellitus: Rat Model Abstract Background. Chronic stress and diabetes mellitus are highly associated with oxidative stress and inflammation, resulting in cell membrane disruption and platelet activity.

This study aims **to** evaluate **the** impact **of chronic psychological stress on the** composition **of** 47

the platelet phospholipid membrane

and platelet activation in type 1 **diabetes mellitus** 66

(T1DM). Methods. We enrolled 35 mature healthy female Wistar rats and

randomly divided them **into 4 groups**, namely the **control group (n = 9**), stress **group (n** = 10), T1DM **group (n** 41

= 8), and T1DM+ Stress group (n = 8). The Wistar rats were treated in different experimental conditions for 28 days while being provided free access to feed and water. The concentration of corticosterone in blood serum and hair samples was measured using a competitive

enzyme-linked immunosorbent assay. Gas chromatography-mass spectrometry 34

was conducted to identify the methyl esters

of fatty acids (FAs) in the platelet phospholipid membrane . A quantitative determination of 1

11-dehydro-thromboxane B2 in the blood serum was also performed using a competitive enzyme-linked immunosorbent assay. Results. After 28 days, the concentration of corticosterone in blood serum (ng/mL) was observed to be higher in the stress group as

compared to the T1DM **and** T1DM+ Stress **groups (P = 0** .031 **and P = 0** .008, **respectively** 13

). The percentage of palmitic FA in the platelet membrane was greater in the T1DM+ Stress group, but its levels of gondoic FA, including alfa-linolenic FA, cervonic FA, and the total sum of omega 3 FAs, were lower as

compared to the control group (P = 0 .016; **P = 0** .016; **P = 0** .031; **P = 0** .016, **P = 0** 6

.031). The concentration of 11-dehydro-thromboxane B2 in blood serum (pg/mL) was observed to be

higher in the stress **group than in** rats with T1DM (**P = 0** 64

.063). Conclusion. chronic psychological stress is related to higher levels of corticosterone, saturated fatty acids in the platelet membrane, and greater platelet activation. This study proves how a low percentage of unsaturated fatty acids in the DM and stress groups indicates the disturbing impact of the oxidative/inflammatory environment to lipid metabolism and neuroendocrine response. Keywords: chronic stress, platelet membrane, fatty acids, diabetes mellitus. Background Nowadays, chronic psychological stress has reached endemic levels across Western countries, contributing to a variety of diseases, like cardiovascular [1], neurodegenerative diseases [2], or endocrine disorders, such as diabetes mellitus (DM) [3]. The activation of physiological stress is mediated by

the hypothalamus-pituitary-adrenal (HPA) axis and the sympathetic nervous system (SNS), leading to the production and **release of glucocorticoids** 35

into systemic circulation [4]. Glucocorticoids (i.e., corticosterone (CORT) in rodents and cortisol in humans), the end-products of the HPA axis, are considered as key players in an organism's response to stress. It is known that stress

hormones not only mediate the stress response, but also regulate metabolism, inflammatory response, and immune function [5]. Since essential fatty acid (FA),

**omega (ω) 3, and ω6 polyunsaturated fatty acids (PUFAs**	1

) provide a foundation for the normal development and functioning

**of the brain and central nervous system** (CNS), **the** development **of**	20

neuropsychiatric disorders is associated with disturbances in FA intake and phospholipid metabolism [6–8]. ω3 PUFAs, mainly

**docosahexaenoic acid** (C **22:6ω3) and eicosapentaenoic acid** (C **20:5ω3**	55

),

**have strong anti-inflammatory, inflammation- resolving**	22

, anti-apoptotic, and anti-oxidative effects. Moreover, ω3 PUFAs antagonize

**the pro- inflammatory effects of** ω6 **PUFAs** , as they **are the precursors of pro- inflammatory mediators**	22

[9]. Disrupted lipid metabolism is also observed in DM, both type 1 and type 2, whose global incidence and prevalence rise dramatically each year [10, 11]. It has been shown that the cellular membranes of 2 diabetics are rich in rigidity-promoting lipids, cholesterol, sphingomyelin, and saturated FAs (SFAs), causing the reduction of membrane fluidity and impaired insulin receptor signaling [12]. Defects in phospholipid membrane composition are also responsible for inflammation processes, resulting in impaired insulin secretion and signaling [12]. Moreover, pro-inflammatory cytokines can indirectly provoke oxidative stress (OS) by activating macrophages, which are known to play a key function in removing the pathogen by generating reactive oxygen species (ROS). As a result, an excess of ROS propagates nonenzymatic lipid peroxidation chain reactions that attack biomembranes [13] and directly damage phospholipids,

resulting in membrane phospholipid oxidation [14]. The increased OS and endothelial activation are related to glucose excursions and directly tied to platelet hyperactivation [15], causing the generation of platelet-derived, highly instable thromboxane A2. The production of TxA2 is a sequential process that begins with arachidonic acid (C 20:4ω6), which is an ω6 PUFA present in the phospholipid membrane. TxA2 is considered as a potent vasoconstrictor of smooth muscles. It promotes platelet activation and aggregation shortly before being hydrolyzed into

11-dehydro-thromboxane B2 (11DHTXB2). As **a stable metabolite** 32

, 11DHTXB2 is a reliable biomarker for platelet activity, which is highly associated with a oxidative/inflammatory environment significant for both DM and chronic psychological stress development and progression [16]. Therefore, this paper aims to determine and evaluate the impact of chronic psychological stress

on the composition of the platelet phospholipid membrane 2

and platelet activation in rats

with type 1 diabetes mellitus (T1DM). This study **provides** insight **into** mitigating **the** effects **of** 10

stress on the physical body, as chronic or prolonged exposure to psychological stress is generally associated with negative health outcomes. Therefore, mitigating the impact of stress is crucial for maintaining overall well-being. Methods Subjects of the study 3 This experimental study involved 35 mature (i.e., 7-month-old 19 subjects and 5-month-old 16 subjects) healthy female Wistar rats (weighing 253 g ± 13.1 g). Animals were randomly housed in cages of 3 rats each under standard conditions (i.e., room temperature 24°C ± 1°C, light/

dark cycle: 12/12 h (light on at 8:00 and light off **at** 50

20:00), relative humidity:

55% ± 5%). The animals had free access to food and water 48

during the experiment. Cages and bedding were replaced weekly. The studied animals were sourced from The Centre for Innovative Medicine. Study design For the first 2 weeks, the animals (n = 35) were subjected to an adaptation period – rats were provided with standard laboratory rodent feed and water ad libitum without causing any stress. After adaptation, the animals were randomly divided into four groups, which were socially housed of three animals per cage and treated in different experimental conditions for 28 days. The rats were weighed on the 1st, 14th, and 28th experimental days, respectively. The first group of rats (n = 9) was a control group that received water and standard feed ad libitum. No intervention was applied to the rodents during the experimental period. Rats from the second group (n = 10) were subjected to chronic psychological stress. Restraint stress was produced daily for two hours

by placing the animal in a small Plexiglas restraint cage **with** 57

sufficient ventilation holes at both ends. No access to food and water was allowed during the procedure [17]. In the third group, rats (n = 8) were used to generate a streptozotocin-induced T1DM state.

A single dose of 65 mg/kg 28

of streptozotocin (MilliporeSigma, USA) treatment was used

to establish diabetes using the procedure described in Furman’s published **protocol** 28

[18] with minor corrections. The rats in the fourth group (n = 8) underwent

a single dose of 65 mg/kg of streptozotocin treatment **to** 28

induce T1DM by Furman’s protocol and were subjected to stress of restraint in small Plexiglas restraint cages. Blood glucose monitoring On the 1st and 28th experimental days, during the first three hours of the light cycle (i.e., to minimize diurnal variation effects), blood glucose levels were determined in all animals using the flavin- dependent glucose dehydrogenase method. Measurements were taken with two CONTOUR®PLUS ONE handheld glucometers (Ascensia Diabetes Care, Basel, Switzerland) using glucose test strips (Lot No. DP0MQHH07B). The utilization of two glucometers ensured accurate and validated blood glucose measurements while reducing the risk of device-related discrepancies. Whole blood was collected by pricking the lateral tail vein with a sterile needle, without the application of local analgesia or anesthesia. The glucometers have a measurement range of 0.6–32.0 mmol/L of glucose in whole blood and an

accuracy of ± 0.5 mmol/L or ± 8.5%, as stated by the manufacturer. Notably, if the glucose level recorded exceeds 32.0 mmol/L, the device will display "HI" instead of providing a numerical result. Thus, such values were recorded as 32.0 mmol/L. Blood glucose monitoring results revealed a statistically significant difference in glucose levels in T1DM and T1DM+Stress groups when comparing the 1st and 28th experimental days (T1DM group: 6.4 (1.95) mmol/L vs 32.0 (1.13) mmol/L, p = 0.016; T1DM+Stress group: 6.8 (0.95) mmol/L vs 32.0 (0.00) mmol/L). The results from the control and stress groups were not significant (control group: 6.3 (0.53) mmol/L vs 6.9 (

1.40) mmol/L, p = 0 42

.322; stress group: 6.4 (0.93)

mmol/L vs 6.3 (**0** .45) **mmol/L** 42

, P = 0.878). Therefore, experimental animals from T1DM and T1DM+Stress groups were considered diabetic. Blood sample collection 5 On the 29th day of the experiment, blood samples were collected from all rats (n = 35) between 10:00 and 12:00 in the morning (i.e., to avoid the peak time of CORT secretion). The collection was done through a cardiac puncture using a 20 G needle. Before the procedure, all rats were anesthetized using a mix of 80% CO2 and 20% O2 [19]. After blood collection, a cervical dislocation was performed to ensure a humane euthanasia of the animal. To determine CORT and 11DHTXB2 concentrations in the blood serum, samples were collected in a 3.5 ml vacutainer tube with an inert gel barrier and clot activator. To analyze the composition of platelet membrane FAs, a 3 ml vacutainer tube with sodium heparin was used to collect the blood samples. Hair sample collection Hair samples (i.e., approximately 150 mg per sample) were collected on days 1 and 29 from all experimental animals. On the 1st day, the rats were shaved to collect their hair samples

from one side of each animal **using an electric razor** 3

without local analgesia or anesthesia.

The shaved area extended from the **dorsal to** the **ventral midline and from neck to tail base** 3

. On day 29, the rats were shaved again to collect

hair samples from the previously shaved sides of 3

each animal after anesthesia and blood collection. Hair samples were wrapped in aluminum foil and

stored in 10 mL polypropylene tubes at 4°C until CORT extraction and analysis . Blood sample preparation After the 3

whole blood was drawn in vacutainer tubes for serum analysis, the samples were allowed to clot

for 15–30 minutes at room temperature. The clot was removed by centrifuging at 1500 **x g for 10 minutes in a refrigerated centrifuge. The** 29

serum samples were apportioned into 0.5 ml aliquots and stored at –80°C 44

. To obtain platelets, a vacutainer tube with an anticoagulant was subjected to centrifugation

at 3000 x g for 10 minutes . Subsequently, three-quarters of the plasma were carefully removed without disturbing the cell and buffy coat layer. The remaining portion, rich in 1

platelets (i.e., one-quarter), was separated,

mixed with freezing media (provided by Biological Industries, Israel) at a ratio of 2:1, and frozen at 2

a temperature of –80 °C. The flow cytometry method and anti-CD31, anti-CD42a antibodies were used to verify the platelet- rich fraction. The result showed that more than 90% of captured cells were of platelets' origin. Hair sample preparation Hair samples were prepared according to the slightly modified protocols published in previous studies [20, 21]. Samples were washed with 5 ml of HPLC-grade 2-isopropanol (Sigma-Aldrich, Germany) for 3 minutes, followed by the removal of the supernatant. Further,

samples were dried in a protected hood 3

at least 24 h at room temperature. Samples consisting of 20 mg of

dried hair were weighed and transferred to 2 mL polypropylene tubes 3

. Then,

1.5 mL of HPLC-grade methanol (Sigma-Aldrich , Germany) was added, **and** 52

the

samples were incubated for 24 h at room temperature **on slow rotation. The** samples **were centrifuged at 10** ,000 g **for 4 min** 3

at 4°C,

and the clear supernatant was transferred into the 2 mL **polypropylene** tubes. **A stream of nitrogen gas was used for the evaporation of methanol and to dry the samples. The dry residue was** resuspended **in** 0.2 mL **of** 16

assay diluent provided in the CORT **enzyme immunoassay kit** 3

. Determination of blood serum and hair corticosterone CORT concentration in serum and hair samples was measured

using a commercially available enzyme immunoassay kit 3

(CORT

competitive ELISA kit, Thermo Fisher Scientific 27

Inc., Carlsbad, CA; Catalog No. EIACORT). The analytical

sensitivity of the assay was 18.6 **pg** /mL, **the intra-assay** 27

CV ranged 7 from 3.1% to 6.5% for the 4

quality controls conducted by the manufacturer 3

. Cross-reactivity was as follows:

desoxycorticosterone 12.30%, tetrahydrocorticosterone 12.30%, **aldosterone 0.62%, cortisol 0.38%, progesterone 0.24%, dexamethasone 0.12** %, < 0.1% **corticosterone-21-hemisuccinate, < 0** .08% **cortisone** and **estradiol** 24

. CORT levels in serum samples are reported in ng/mL, and in hair samples – pg/mg. Determination of platelet membrane fatty acids After allowing the samples to thaw at room temperature, the Folch method [22] was used to extract the lipids from the platelet membrane. Subsequently,

thin-layer chromatography was performed (**Sil G-25 UV 254) to** separate the **platelet** membrane **phospholipids [23**]. Following **transesterification** 1

,

gas chromatography-mass spectrometry was conducted using **a GCMS-QP2010 Ultra (Shimadzu** 1

, Japan) analyzer to identify the methyl esters of FAs. The obtained

data were then collected and processed using LabSolutions (Shimadzu 1

, Japan) software version 4.52.

The content of each FA in the total FA amount (100 2

%) was determined by calculating

the percentage of SFAs (i.e., C 14:0, C 16:0, C 18:0), monounsaturated FAs (MUFAs) (i.e., C 16:1ω7, C 18:1ω9, C 18:1ω7, C 20:1ω9), PUFAs (i.e., C 18:2ω6, C 18:3ω3, C 20:4ω6, C 20:5ω3, C 22:5ω3, C 22:6ω3), and the percentage of PUFAs ω3 and ω6 2

. Determination of blood serum 11-dehydro thromboxane B2 A quantitative determination of rat 11DHTXB2 in blood serum was performed

using a commercially available enzyme immunoassay kit 3

(11DHTXB2 competitive ELISA kit, MyBioSource Inc., San Diego, CA; Catalog No. MBS289362).

The sensitivity of the assay was 1 .0 **pg** /mL. CV in **the** same lot **and** 27

a different lot was less than 10% according to the manufacturer. No significant cross-reactivity or interference between 11DHTXB2 and an analogue was observed. Levels of blood serum 11DHTXB2 are presented in pg/mL.

Statistical analysis The **statistical analysis was carried out using** the **R Studio** and **R** 51

Commander software packages. MS Excel 2019 was used for visual representation. As the total number of subjects was 35, nonparametric tests were performed. For more precise correlations between

**the composition of platelet membrane FAs** , the concentrations **of** hair and **blood serum**	1

CORT, and 11DHTXB2, nonparametric Spearman's correlation coefficient was applied. This study presents data in the median, minimum, and maximum values, along with the interquartile range. The

**Kruskal-Wallis** test **was used to determine differences in the**	24

median values of distinct biomarkers between the four experimental groups. The Mann-Whitney-Wilcoxon

**test was used as a post hoc test**	39

to determine the differences between each of the

**groups. Statistical significance was determined by a P-value** below **0.05. Results**	46

Body weight Animal body weight increased by 2.2 percent in the stress group comparing the 14th and 28th experimental days (273 (13.5) g vs 279 (16.5) g, P = 0.009) and by a total 3.3 percent increase comparing the 1st and 28th experimental days (270 (17.8) g vs 279 (16.5) g P = 0.040). Rats from T1DM and T1DM+Stress groups showed a statistically significant decrease of body weight. A total 9.8 percent weight reduction was observed in the T1DM group when comparing the 1st and 28th experimental days (246 (9.5) g vs 222 (16.0) g, P = 0.016); a 8.9 percent of total weight reduction was reached during the first 14 days (246 (9.5) g vs 224 (17.0) g, P = 0.031). By comparing rats' body mass in the 9 T1DM+Stress group between the 1st and 14th days, a clear 17.5 percent weight loss (240 (10.0) g vs 198 (29.0) g, P = 0.014) and a total 14.5 percent mass reduction was observed between 1st and 28th experimental days (240 (10.0) g vs 205 (33.0) g, P = 0.014). In the same group, was a slight 3.0 percent increase of body weight during the last fourteen days (198 (29.0) g vs 205 (33.0) g, P = 0.269). Rats from the control group also had a total 5.5 percent increase of body mass (256 (7.0) g vs 270 (11.8) g).

**The results** , however, **were not statistically significant (P = 0**	63

.253). Blood serum and hair corticosterone Blood serum CORT concentration (Table 1) was significantly lower in T1DM and in T1DM+Stress groups as

compared to the stress **group (P = 0.031, P = 0** .008). **Rats with** 31

T1DM also had a lower concentration of CORT in their

blood serum than those in the control group (P = 0 .031). However, **the** concentration **of** 2

CORT in hair samples was statistically

significantly higher in the stress **group when** comparing **the** 1st **and** 17

29th experimental days (P = 0.027) (Fig. 1); likewise, the concentration of CORT in their blood serum was statistically significantly higher when compared to other experimental groups. However, the results of hair CORT in T1DM and in T1DM+Stress groups showed no significant difference. Table 1 A comparison of blood serum corticosterone concentrations among different experimental groups Blood serum (Units) Median, minimum, maximum, interquartile range Control group (n=9)1 Stress group (n=10)2 T1DM group (n=8)3 T1DM+Stress group (n=8)4 P-values* Corticosterone Minimum 16.02 28.08 51.26 56.28 1,4 = 0.219 Median 326.10 549.30 149.45 126.65 1,2 = 0.148 1,3 = 0.031 (ng/mL) Maximum 632.90 899.40 515.20 640.90 2,3 = 0.031 2,4 = 0.008 IQR 95.25 307.91 174.57 43.81 3,4 = 1.00 215 T1DM – type 1 diabetes mellitus, IQR – interquartile range. * Mann-Whitney-Wilcoxon test Platelet membrane FA composition The composition of FAs in platelet phospholipid membranes is listed in Table 2. An analysis of SFAs

showed that the level of C 14:0 **was** lower **in the** 1

stress and in T1DM+Stress groups as compared to control (P = 0.023, P = 0.047), but C 16:0 was

observed to be higher in the T1DM+Stress **group (P = 0** 21

.016)

as compared to the control group . Rats from **the** same experimental **group** 6

(i.e., T1DM+Stress group) also

had a higher level of C 16: 0 as compared to the stress group (P = 0	1

.055) (Fig. 2), but their level of C 18:0 was lower (P = 0.008). Moreover, C 18:0 of platelet phospholipid membrane

decreased in the T1DM group as compared to stress group rats (P = 0.031	31

). By analyzing the results of MUFAs in platelet phospholipid membranes, less

C 18:1ω7 and C 20 :1ω9 were observed in the	60

T1DM+Stress

group than in the control rat group (P = 0 .047; P = 0	9

.016). Furthermore, the tendency of a lower level of C 20:1ω9 was also

observed in the T1DM+Stress group, when comparing to the stress group (P	36

= 0.068) and the T1DM group separately (P = 0.036) (Fig. 3). The total sum of ω3 PUFAs was lower in all intervention groups than in control animals. Moreover, the lowest level of total ω3 PUFAs was in T1DM+Stress group compared to control (P = 0.031) (Fig. 4, panel A). The percentages

of C 18:3ω3 and C 20:5ω3 PUFAs also	2

were

statistically significantly lower in platelet phospholipid membranes of	5

the T1DM+Stress group as

**compared to control** rats (**P = 0.031; P = 0**	13

.016). The results

**of C 20:5ω3 and C 22**	5

:5ω3 (Fig. 4, panel B and C) showed a decreasing level tendency in rats with T1DM

**compared to the control group (P = 0.031, P = 0**	13

.093). A similar tendency was observed for C 22:5ω3 in the stress group in comparison with control (P = 0.079). However, experimental rats from the stress group had a higher percentage of

**C 22:6ω3 in their platelet phospholipid** membranes **than** rats from **the** T1DM+Stress **group** (P = **0**	5

.016). Blood serum 11-dehydro thromboxane B2 When examining the results of the 11DHTXB2, a consistent trend of decreasing concentrations was observed among all the experimental rat groups

**as compared to the control group** , as depicted in **Fig**	39

. 5. Rats with T1DM exhibited reduced levels of 11DHTXB2 in their blood serum when

**compared to** both **the** control **and** stress **groups** individually (**P = 0** .094; **P = 0**	13

.063). Correlation results In stress group, blood serum CORT showed a correlation with C 18:3ω3 (

r = -0 .6280605, **P = 0** .052) **and** 11DHTXB2 (**r = -0** .6848485, **P = 0** 11

.035).

A moderate direct **correlation between C 18:0 and** 11DHTXB2 (**r = 0** 2

.6363636, P = 0.054) in the stress group was observed as well (

r = 0 .6707442, **P = 0** .034). **There was also a** moderate direct **correlation between** C 18:3ω3 **and** 8

C 20:5ω3

in the T1DM **group (r = 0** .682647, **P = 0** 36

.062),

and between C 18 :3ω3 **and C** 22:5ω3 (**r = 0** .7831325, P = **0** 54

.022) in the T1DM+Stress group. Discussion According to our study results, rats in the restraint stress group (i.e., a simulation for chronic psychological stress such as occupation-related stress in humans [17]) had the highest level of hair and blood serum CORT as compared to other experimental groups. Moreover, rats in the stress group had a significant weight gain compared with the other groups. Such results were expected, as it is widely known that stress has a major effect on metabolic activity and stimulates the release of various hormones. Increasing the concentration of CORT stimulates insulin secretion in nondiabetic rats and humans. The high level of glucocorticoids, accompanied by a high concentration of insulin, exerts a strong anabolic effect on fat, particularly abdominal fat, resulting in weight gain [24, 25]. However, rats in the T1DM and in T1DM+Stress groups clearly demonstrated lower concentrations of blood serum CORT followed by a significant decrease of body mass. These results could likely be attributed to various factors, including habituation, fatigue, or a combination of both. According to the scientific 12 data, long-term hypokinesia or repeated stress may lead to an absence of the adrenal response; thus, rats may elevate their CORT levels mostly to new stimuli. It must also be noted that generally, physical stress induces a greater hormonal response than psychological

stress. Secondly, when the stimuli are more severe and weight loss occurs (mesenteric and other fat stores decrease), due to induced metabolic disorder (i.e., reduction of insulin secretion) and greater sensitivity, rodents may develop an exhaustion (i.e., the state when an animal can no longer compensate and their condition becomes life-threatening) [26, 27]. Additionally, CORT, the primary glucocorticoid produced in the adrenal cortex of rats, may decrease due to the loss of body fat, which serves as the initial substrate for the synthesis of steroid hormones [28]. By analyzing the platelet membrane phospholipid FA results acquired in the present study an increase of SFA (

i.e., C 16:0, C 18:0 1

) was observed in rats experiencing chronic psychological stress as

compared to the control group. Similar results were 62

published in Bernardi's et al. paper [29]. Its authors assessed an early stressful event, such as the maternal separation of Wistar rat litters, and interaction with the nutritional availability of ω3 PUFAs during the life course. Their data showed that maternal- separated rats with an adequate diet of ω3 PUFAs had a higher level of SFAs in their peripheral blood than non-handled rats with the same diet. Moreover, maternal separated rats had an increased abdominal fat deposition resulting in body weight gain compared to the non-handled ones. A likewise tendency was observed in our study. Hennebelle et al. [30] also noticed similar results in their research, analyzing FAs in the plasma of rats fed with a control diet and having repeated restraint stress as compared to rats with the same diet and no stress. These rats had a plasma CORT increase, as the one observed in our study. Scientific data note that elevated plasma-free FAs correlate with certain psychological alterations. SFAs, especially C 16:00, induce

anxiety-like behavior while increasing amygdala-based serotonin metabolism 38

. Furthermore, C 16:00 has the potential to

be lipotoxic to cells by causing the **accumulation of** 38

ROS, which can result in endoplasmic reticulum (ER) stress and 13 ultimately lead to cell apoptosis in both humans and model organisms, as described by Moon et al. [31]. ER, consequently, can result in cellular dysfunction and diseases such as neurodegeneration and DM [32]. Our experimental animals with T1DM and T1DM+Stress showed a clear

decrease of

C 14:0, C 18:0 and an increase of **C** 16: **0** 56

accompanied by a significant body weight loss. The parallel results were observed

in a study by Shen **et al** . [33], which analyzed **the composition of** 59

plasma FAs in healthy rats and in rats with T1DM induced by streptozotocin. Moreover, patients with T1DM and bad glycemic control also had similar SFA results in their plasma as compared to healthy controls

in a study by Sobczak **et al** . [34]. Elevated levels **of** the **total** 18

sum of SFAs in the T1DM groups could be due to SFA C 16:0 serving as an energy source or a building block for lipid metabolism, as well as its pathogenic roles: working

as a signaling molecule that regulates **the progression and development of many diseases at the molecular level** 4

. Recent scientific studies demonstrate that a high level of plasma C 16:0 increases the cellular uptake of C 16:0, leading to insulin signaling inhibition and

the development of insulin resistance. Furthermore, in pancreatic islets , C 16:0 **inhibits glucose-induced insulin secretion by impairing exocytosis evoked by action potential-like depolarization** 4

. SFA C 16:0

also increases the production of interleukin (IL) 1β that not only promotes the development of insulin resistance in tumor necrosis factor (TNF)-dependent and TNF-independent pathways, but also activates autophagy by inducing ER stress 4

and causing metabolic dysregulation [35]. Our study showed a lower percentage of MUFAs in all intervention groups as

compared to the control group. The data **of** decreased MUFAs **in** 6

plasma were also noticed in rats with repeated restraint stress [30]. Moreover, Juncker et al. [36] studied the FA composition of human milk in women experiencing postpartum stress. They noticed that women who experienced higher levels of stress had a significantly lower levels of MUFAs in mature milk than women in a control group. There is increasing evidence linking MUFAs to an anti-inflammatory effect, as they may

lower levels of circulating mononuclear 14 **cells** (i.e., **monocytic cells** are **involved in the inflammatory response** 40

), increase circulating

anti- inflammatory markers (IL-4 and IL-10), and lower proinflammatory markers (**IL** 49

-6, monocyte chemoattractant protein-1, IL-1 and TNF-α). In addition, MUFAs can generate a more favorable plasma lipid profile and increase cognitive function [37]. Thus, we assume that our experimental animals with declined MUFA levels might have underwent an inflammatory status leading to increased MUFAs oxidation, as they were more sensitive to peroxidation than SFAs. The same explanation could be applied to T1DM and T1DM+Stress rats, as low-grade inflammation has also been associated with DM. Furthermore, it is known that MUFAs are related to anti-diabetic effects by improving

the insulin receptor substrate 1/phosphoinositide 3-kinase 30

insulin signaling pathway, activating the

adenosine monophosphate-activated protein kinase, and 30

translocating glucose transporter 4. The anti-hyperglycemic effect is also observed, as MUFAs may enhance insulin sensitivity and glucose uptake, and they may inhibit hepatic gluconeogenesis or other relevant insulinotropic actions [38]. Therefore, we support the hypothesis that the abovementioned processes could have been altered in our experimental subjects due to a lack of MUFAs, which was also observed in other DM animal studies [39, 40]. Scientific data demonstrate that PUFAs, especially ω3

PUFAs, have a wide range of effects on mental health **at the molecular and cellular levels** 22

, as they optimize

membrane fluidity and lipid bilayer elasticity 22

. ω3 PUFAs improve ion channel function and the binding of neurotransmitters and their receptors in the membrane, stimulate the expansion of cell membranes at the nerve growth cones, and regulate the activity of signal molecules, gene expression, and epigenetic modifications. Moreover, ω3 PUFAs may antagonize inflammation and modulate the immune response, affect mitochondrial function and ROS homeostasis, cell proliferation, viability and cell repair, or apoptosis [9]. However,

epidemiological and animal studies show **a negative correlation between** the **status** 33

of ω3 PUFAs

and stress-associated disorders, such as anxiety and depression 33

. Laurego et al. [41] demonstrated in their 15 study that chronically stressed titi monkeys (C. cupreus) had a lower plasma ω3 FA status than the control group. Larrieu et al. [42] also noticed that a deficiency of dietary ω3 PUFAs induces the chronic stress phenotype in mice. Moreover, they

measured HPA axis activity and found that an ω3 **deficiency induced a significant increase in plasma** CORT **levels in undefeated** ω3- **deficient mice** as **compared** to **undefeated control diet mice. Similar** results were obtained **in** 26

our study, where chronically restrained rats had a lower level of ω3 PUFAs, a higher concentration of hair and blood serum CORT, and a clearly negative correlation between C 18:3ω3 and blood serum CORT. Moreover, the results showed that the lower C 18:3ω3 (i.e., the main substrate of ω3 long-chain PUFA biosynthesis) percentage gets, the lower level of C 20:5ω3 is detected. This clearly indicates that the organism may develop a disrupted lipid metabolism due to overuse of chronic stress. The levels of total ω3 PUFAs and certain ω3 FAs (i.e.,

C 18:3ω3, C 20:5ω3, C 22:5ω3 19

) also decreased in T1DM and T1DM+Stress rats

as compared to the control group in the present **study** . Moreover, the decline **in** percentage **of** 34

C 18:3ω3 resulted in decreased levels of

C 20:5ω3 and C 22 :5ω3 **in the** T1DM **and** 58

T1DM+Stress groups, respectively. Yao et al. [43] demonstrated similar results: DM rats had a lower level of C 18:3ω3,

C 20:5ω3, and C 22 :5ω3 PUFAs **in their** liver samples **than** those of **the control group** 5

. Krishna Mohan and Das [44] found a significantly lower level of plasma PUFAs in alloxan-induced T1DM compared to an untreated group. Studies show that free-radical generation is increased in diabetic animals and in patients with T1DM and type 2 DM. Moreover, pancreatic

antioxidant enzymes, such as superoxide dismutase , glutathione peroxidase, and **catalase** 30

, exhibit less activity, which leads to enhanced formation of free radicals in diabetes. Therefore, these processes might accelerate lipid peroxidation [44]. Moreover, it is known that FA desaturases (i.e., Δ5- and Δ6- desaturase) are the key enzymes for the biosynthesis of PUFAs. The absence of insulin in animal models and in T1DM patients results in a marked decrease in the biosynthesis of PUFAs, including C 20:4ω6, because of the lower transcription and lower activity of Δ5- and Δ6-desaturases [43]. Therefore, declining levels of ω3 PUFAs could be observed. DM is associated with OS due to intracellular hyperglycemia, or an increased oxidation of FAs and superoxide, and low antioxidant capacity. Moreover, low-grade

inflammatory stimuli also **induce** an **increased lipid peroxidation with consequent platelet activation, resulting in** 43

thromboxane A2 (TxA2) and further OS [15]. Enzymatically produced TxA2 from C 20:4ω6 is

hydrolyzed into a biologically inactive but more stable thromboxane B2 (TxB2) metabolite. TxB2 32

is further metabolized primarily into a 11DHTXB2 form [16]. According to scientific data, 11DHTXB2 is a marker of platelet activity and inflammation, which increases in DM subjects, both humans and experimental animals [15, 45]. However, the findings of this study demonstrate the contrary: all experimental groups had lower levels of 11DHTXB2 as compared to control rats, although the stress group had the highest concentration among other affected groups. The explanation for this could probably be traced to an imbalance between the production and accumulation of ROS, a common factor in DM and chronic stress, leading to lipid peroxidation and a declined activity of the aforementioned desaturases. Therefore, decreasing levels of C 20:4ω6 in rat platelet phospholipid membranes, where C 20:4ω6 is the main precursor of 11DHTXB2 biosynthesis, might be observed. The decrease was especially evident in the present study, which recorded a negative correlation between blood serum CORT and 11DHTXB2 of the stress group. Study strengths and limitations

The main strength **of this study was** that **the** relationship between **chronic** 12

psychological stress, alteration in platelet membrane FA, and increased platelet activation in T1DM was evident, although experimental subject groups were small. In addition, the model of chronic stress chosen for the experimental

animals simulated occupation-related stress, which is considered one of the most common chronic stressors in humans. 17 There were also a few potential limitations in the present study. First, it measured a biomarker of inflammation, but there was no evaluation of oxidative stress and/or activation of antioxidant enzymes, even though both DM and chronic psychological stress undergo such conditions simultaneously. Second, we did not consider applying insulin and/or a reduction of chronic psychological stimuli, or even certain FAs, as treatment options in order to get the reverse outcome, though scientific data declare that most of the abnormalities revert to normal conditions by applying the aforementioned treatment.

**Conclusions The findings of the present study** indicate **that** chronic psychological **stress**	15

leads to increased production of the primary stress hormone, CORT, and heightened platelet activation within the systemic circulation. Additionally, the percentage of SFAs, particularly C 16:00, was found to be elevated

**in the platelet phospholipid membrane** both **in** the stress **and**	5

T1DM groups. The levels of MUFAs (i.e.,

**C 18:1ω7, C 20:1ω9**) and **PUFAs**	5

, especially ω 3 FAs (i.e.,

**C 18:3ω3, C 20:5ω3, C 22:5ω3, C 22:6ω3**	19

), were lower in chronically stressed animals. This clearly indicates the disruptive impact of chronic stress and DM to lipid metabolism and the neuroendocrine response. These processes are highly related to the inflammatory and oxidative status in the animal model; they can induce a hypercoagulable state, leading to the development and progression of cardiovascular outcomes. Abbreviations DM

**Diabetes mellitus HPA Hypothalamus-pituitary-adrenal axis SNS Sympathetic nervous system**	7

CORT Corticosterone FA Fatty acid Ω Omega PUFAs Polyunsaturated fatty acids CNS Central nervous system

SFAs Saturated fatty acids ROS Reactive oxygen species 17

11DHTXB2 11-dehydro-thromboxane B2 T1DM Type 1 diabetes mellitus MUFAs Monounsaturated fatty acids ER Endoplasmic reticulum

IL Interleukin TNF Tumor necrosis factor Acknowledgments The authors would like **to** 53

thank all researchers who contributed to

this study. **Authors' Contributions** IB, JB, RR, **and** DK **contributed to the** conception and **design of the study** 45

.

IB wrote the first draft of the manuscript 61

. EM, JB, RR, RV, RŠR, AK, and DK contributed by reviewing and editing the daft. IB, JB, RR, EM, and DK contributed to the formal analysis and interpretation

of the results. **All authors** have **contributed to** the paper **and approved the submitted version.** **Funding** 19 **This** study **was** supported **by** Vilnius University and **the** 37

Centre for Innovative Medicine. Data Availability

The datasets used and/or analyzed during the current study are available from the corresponding author on reasonable request. Declarations Ethics approval and consent to participate The study was approved by the 23

State Food and Veterinary Service (Approval No. G2–217).

Consent for publication Not applicable. Competing interests The authors declare that the research was conducted in the absence of any commercial or financial relationships that could be construed as a potential conflict of interest . Figures Fig. **1** A comparison **of** 14

hair corticosterone concentrations in the animal stress group. * P = 0.027, n = 10 Fig. 2 A comparison of C 16:0 percentages in the platelet membrane across different experimental groups. * P = 0.016, ** P = 0.055 Fig. 3 A comparison

of C 20 :1ω9 percentages **in the platelet membrane** 5

across different experimental groups. *

P = 0 .016, ** **P = 0** .068, *** **P = 0** .036 20 440 **Fig. 4** 65

A comparison of ω3 PUFAs in platelet membrane across different experimental groups. (A) 441 shows the percentage of total ω3, * P = 0.0313; (B) shows the percentage of C 20:5ω3, * P = 0.031, 442 ** P = 0.016; (C) shows the percentage of C 22:5ω3, * P = 0.079, ** P = 0.093 443 Fig. 5 A comparison of blood serum 11-dehydro thromboxane B2 concentrations across different 444 experimental groups. * P = 0.094; ** P = 0.063 445

Table 2 A comparison of the composition of FAs **in** 2

rats' platelet membranes across different 446 experimental groups. Platelet

FAs (provided by percentage of total amount) Median, minimum, maximum , interquartile range **Control group (n** 2

=9)1 Stress group (n=10)2 T1DM group (n=8)3 T1DM+Stress group (n=8)4 P-values Minimum 2.95 1.98 1.64 1.40 C 14:0* 1,4 = 0.047 Median 4.77 3.47 2.60 1.87 1,2 = 0.023 1,3 = 0.219 $ Maximum 6.56 4.68 6.45 5.84 2,3 = 1.000 2,4 = 0.383 IQR 0.88 0.81 1.08 1.33 3,4 = 0.563 C 16:0 Minimum 43.83 46.66 47.68 49.30 1,4 = 0.016 Median 51.40 52.43 55.15 57.66 1,2 = 0.742 1,3 = 0.156 $ Maximum 54.38 55.2 57.58 69.44 2,3 = 0.563 IQR 4.63 4.29 2.96 6.53 2,4 = 0.055 3,4 = 0.313 Minimum 31.39 33.64 27.82 20.06 C 18:0 1,4 = 0.297 Median 38.41 39.60 37.35 34.50 1,2 = 0.195 1,3 = 0.313

$ Maximum 42.85 43.62 46.54 38.43 2,3 = 0.031 IQR 6.19 2.95 4.54 9.46 2,4 = 0.008 3,4 = 0.844 Median 0.69 0.30 0.23 0.52 C 16:1ω**7 Minimum 0.39 0.11 0.14 0.03 0.199# Maximum 3.07 1.26 2.19 1.27 IQR 0.76 0.50 0.48 0.67 C 18:1ω7 Minimum 0.10 0.04 0.06 0.21 1,4 = 0.047 Median 0.51 0.34 0.20 0.39 1,2 = 0.461 1,3 = 0.313 $ Maximum 1.78 1.54 1.35 0.85 2,3 = 0.313 2,4 = 0.945 IQR 0.83 0.78 0.33 0.31 3,4 = 0.916 Median 1.70 1.03 0.81 1.40 C 18:1ω9 Minimum 0.43 0.10 0.19 0.31 0.568# Maximum 4.35 2.81 4.37 3.42 IQR 1.19 1.45 1.04 0.75 C 20:1ω9 Minimum 0.19 0.14 0.11 0.12 1,4 = 0.016 Median 0.31 0.26 0.24 0.17 1,2 = 0.148 1,3 = 0.156 $ Maximum 0.59 0.41 0.42 0.21 2,3 = 0.438 2,4 = 0.068 IQR 0.16 0.17 0.07 0.04 3,4 = 0.036 Median 1.91 1.205 1.55 2.00 C 18:2ω6 Minimum 0.33 0.43 0.65 0.72 0.580# Maximum 5.55 4.94 8.20 8.02 IQR 1.36 1.61 1.00 1.19 C 18:3ω3 Minimum 0.43 0.17 0.13 0.11 1,4 = 0.031 Median 0.59 0.32 0.28 0.25 1,2 = 0.148 1,3 = 0.400 $ Maximum 0.82 0.92 1.03 0.92 2,3 = 0.313 2,4 = 0.483 IQR 0.20 0.15 0.25 0.25 3,4 = 0.688 Median 0.49 0.33 0.28 0.19 C 20:4ω6 Minimum 0.10 0.06 0.10 0.13 0.285# Maximum 1.94 1.39 0.6 1.08 IQR 0.42 0.54 0.20 0.26 Median 0.29 0.12 0.08 0.06 1,2 = 0.195 $ C 20:5ω3 Minimum 0.09 0.02 0.02 0.01 1,3 = 0.031 1,4 = 0.016 Maximum 0.40 0.38 0.21 0.25 2,3 = 0.787 IQR 0.09 0.09 0.06 0.04 2,4 = 0.262 3,4 = 0.563 Minimum 0.05 0.03 0.03 0.01 C 22:5ω3 1,4 = 0.375 Median 0.08 0.06 0.07 0.06 1,2 = 0.079 1,3 = 0.093 $ Maximum 0.14 0.09 0.15 0.14 2,3 = 0.343 IQR 0.06 0.04 0.06 0.06 2,4 = 0.554 3,4 = 0.590 C 22:6ω3 Minimum 0.02 0.04 0.03 0.02 1,4 = 0.178 Median 0.07 0.07 0.08 0.05 1,2 = 1.000 1,3 = 1.000 $ Maximum 0.12 0.13 0.16 0.07 2,3 = 0.844 IQR 0.05 0.05 0.07 0.02 2,4 = 0.016 3,4 = 0.156 Median 92.80 95.77 96.29 94.32 Σ*** SFAs Minimum 81.63 87.27 83.01 85.21 0.428# Maximum 96.89 98.63 97.3 97.71 IQR 3.79 5.57 2.90 3.10 Median 3.64 1.89 1.31 2.59 Σ MUFAs Minimum 1.41 0.41 0.93 0.73 0.420# Maximum 9.71 5.73 7.78 5.23 IQR 2.32 2.87 1.85 1.71 Median 3.57 2.32 2.48 3.03 Σ PUFAs Minimum 1.70 0.96 1.56 1.57 0.440# Maximum 8.66 7.01 9.24 9.56 IQR 1.48 2.71 1.02 1.68 Σ ω3 Minimum 0.70 0.29 0.24 0.19 1,4 = 0.031 Median 1.07 0.60 0.60 0.42 1,2 = 0.109 1,3 = 0.156 $ Maximum 1.41 1.45 1.31 1.31 2,3 = 0.313 IQR 0.17 0.24 0.25 0.32 2,4 = 0.195 3,4 = 0.438 Median 2.45 1.53 1.86 2.18 Σ ω6 Minimum 0.43 0.49 0.78 0.85 0.597# Maximum 7.49 6.33 8.8 9.1 IQR 1.90 2.19 1.15 1.48 447

SFAs – saturated fatty acids, MUFAs – monounsaturated fatty acids, PUFAs – polyunsaturated fatty acids 1

, T1DM – type 448 1 diabetes mellitus, FAs – fatty acids, IQR –

interquartile range, * – number of carbon atoms and double bonds, ** – position 449 **of double bond between carbon atoms in the molecule, *** – total sum** 1

, $Mann-Whitney-Wilcoxon test, #

Kruskal-Wallis test. 1 2 3 4 5 6 7 8 9 10 11 12 13 14 15 16 17 18 19 20 21 22 23 24 25 25

26 27 28 29 30 31 32 33 34 35 36 37 38 39 40 41 42 43 44 45 46 47 48 49 50 51 52 53 54 55 56 57 58 59 60 61 62 63 64 65 66 67 68 69 70 71 72 73 74 75 76 77 78 79 80 81 82 83 84 85 86 87 88 89 90 91 92 93 94 95 96 97 98 99 100 101 102 103 104 105 106 107 108 109 110 111 112 113 114 115 116 117 118 119 120 121 122 123 124 125 126 127 128 129 130 131 132 133 134 135 136 137 138 139 140 141 142 143 144 145 146 147 148 149 150 151 152 153 154 155 156 157 158 159 160 161 162 163 164 165 166 167 168 169 170 171 172 173 174 175 176 177 178 179 180 181 182 183 184 185 186 187 188 189 190 191 192 193 194 195 196 197 198 199 200 201 202 203 204 205 206 207 208 209 210 211 212 213 214 216 217 218 219 220 221 222 223 224 225 226 227 228 229 230 231 232 233 234 235 236 237 238 239 240 241 242 243 244 245 246 247 248 249 250 251 252 253 254 255 256 257 258 259 260 261 262 263 264 265 266 267 268 269 270 271 272 273 274 275 276 277 278 279 280 281 282 283 284 285 286 287 288 289 290 291 292 293 294 295 296 297 298 299 300 301 302 303 304 305 306 307 308 309 310 311 312 313 314 315 316 317 318 319 320 321 322 323 324 325 326 327 328 329 330 331 332 333 334 335 336 337 338 339 340 341 342 343 344 345 346 347 348 349 350 351 352 353 354 355 356 357 358 359 360 361 362 363 364 365 366 367 368 369 370 371 372 373 374 375 376 377 378 379 380 381 382 383 384 385 386 387 388 389 390 391 392 393 394 395 396 397 398 399 400 401 402 403 404 405 406 407 408 409 410 411 412 413 414 415 416 417 418 419 420 421 422 423 424 425 426 427 428 429 430 431 432 433 434 435 436 437 438 439 4 6 8 10 11 16 18 21 22

sources:

1 141 words / 2% - Internet from 27-Jan-2023 12:00AM
epublications.vu.lt

2 137 words / 2% - Internet from 16-Mar-2022 12:00AM
epublications.vu.lt

3 109 words / 1% - Crossref
Rebecca L. Erickson, Caroline A. Browne, Irwin Lucki. "Hair corticosterone measurement in mouse models of type 1 and type 2 diabetes mellitus", Physiology & Behavior, 2017

4 72 words / 1% - Internet from 31-Jan-2020 12:00AM
link.springer.com

5 58 words / 1% - Crossref
Inga Bikulčienė, Neda Garjonytė, Vytautas Žėkas, Rėda Matuzevičienė et al. "Relationship Between Composition of Fatty Acid in Platelet Phospholipid Membrane and Markers of Oxidative Stress in Healthy Men and Men After a Myocardial Infarction", Medical Science Monitor Basic Research, 2021

6 31 words / < 1% match - from 23-Apr-2023 12:00AM
www.science.gov

7 11 words / < 1% match - from 11-Jun-2023 12:00AM
www.science.gov

8 11 words / < 1% match - Internet from 14-Jan-2023 12:00AM
www.science.gov

9 10 words / < 1% match - Internet from 13-Jan-2023 12:00AM
www.science.gov

10 9 words / < 1% match - from 28-Sep-2023 12:00AM
www.science.gov

11 9 words / < 1% match - from 29-May-2023 12:00AM
www.science.gov

12 8 words / < 1% match - from 29-Jun-2023 12:00AM
www.science.gov

13 38 words / < 1% match - from 18-Nov-2023 12:00AM
www.researchsquare.com

14 36 words / < 1% match - from 19-Oct-2023 12:00AM
www.researchsquare.com

15 9 words / < 1% match - from 13-Sep-2023 12:00AM
www.researchsquare.com

16 32 words / < 1% match - Internet from 10-Oct-2022 12:00AM
www.mdpi.com

17 16 words / < 1% match - Internet from 02-May-2020 12:00AM
www.mdpi.com

18 8 words / < 1% match - Internet from 14-Mar-2020 12:00AM
www.mdpi.com

19 21 words / < 1% match - Internet from 18-Dec-2022 12:00AM
worldwidescience.org

20 9 words / < 1% match - Internet from 17-Oct-2021 12:00AM
worldwidescience.org

21 9 words / < 1% match - from 24-Dec-2023 12:00AM
worldwidescience.org

22 38 words / < 1% match - Crossref
Lie Zhou, Jia-Yao Xiong, Yu-Qian Chai, Lu Huang, Zi-Yang Tang, Xin-Feng Zhang, Bo Liu, Jun-Tao Zhang. "Possible antidepressant mechanisms of omega-3 polyunsaturated fatty acids acting on the central nervous system", Frontiers in Psychiatry, 2022

23 32 words / < 1% match - from 19-Dec-2023 12:00AM
asja.springeropen.com

24 31 words / < 1% match - Internet
"Ecology and Conservation of Parrots in Their Native and Non-Native Ranges", 'MDPI AG', 2022

25 28 words / < 1% match - Internet from 10-Dec-2022 12:00AM
randr19.nist.gov

26 26 words / < 1% match - Crossref
Larrieu, T, L M Hilal, C Fourrier, V De Smedt-Peyrusse, Sans N, L Capuron, and S Layé. "Nutritional omega-3 modulates neuronal morphology in the prefrontal cortex along with depression-related behaviour through corticosterone secretion", Translational Psychiatry, 2014.

27 25 words / < 1% match - Crossref
Alberto Elmi, Viola Galligioni, Nadia Govoni, Martina Bertocchi et al. "Quantification of Hair Corticosterone, DHEA and Testosterone as a Potential Tool for Welfare Assessment in Male Laboratory Mice", Animals, 2020

28 24 words / < 1% match - Crossref
Kenneth K. Wu. "Streptozotocin-Induced Diabetic Models in Mice and Rats", Current Protocols in Pharmacology, 03/2008

29 24 words / < 1% match - Internet from 08-Mar-2019 12:00AM
dspace.bracu.ac.bd

30 21 words / < 1% match - Crossref
Wei Ding, Huimin Liu, Ziqi Qin, Meihong Liu, Mingzhu Zheng, Dan Cai, Jingsheng Liu. "Dietary Antioxidant Anthocyanins Mitigate Type II Diabetes through Improving the Disorder of Glycometabolism and Insulin Resistance", Journal of Agricultural and Food Chemistry, 2021

31 21 words / < 1% match - from 27-Jun-2023 12:00AM
apo.ansto.gov.au

32 21 words / < 1% match - Internet from 17-Jul-2016 12:00AM
www.wjgnet.com

33 18 words / < 1% match - Crossref
Marie Hennebelle, Gaëlle Champeil-Potokar, Monique Lavialle, Sylvie Vancassel, Isabelle Denis. "Omega-3 polyunsaturated fatty acids and chronic stress-induced modulations of glutamatergic neurotransmission in the hippocampus", Nutrition Reviews, 2014

34 18 words / < 1% match - Internet from 28-Jan-2023 12:00AM
www.researchgate.net

35 17 words / < 1% match - Internet from 04-Mar-2014 12:00AM
www.dataprevproject.net

36 16 words / < 1% match - Crossref
"Book of Abstracts", Climacteric, 2009

37 16 words / < 1% match - ProQuest
McCabe, Tempest. "Modeling Ecological Disturbances in the Southeastern United States", Boston University, 2023

38 15 words / < 1% match - Crossref
Morgan L. Moon, Jennifer J. Joesting, Marcus A. Lawson, Gabriel S. Chiu, Neil A. Blevins, Kristin A. Kwakwa, Gregory G. Freund. "The saturated fatty acid, palmitic acid, induces anxiety-like behavior in mice", Metabolism, 2014

39 15 words / < 1% match - ProQuest

Márványkövi, Fanni Magdolna. "Investigation of the Pathomechanism of Uremic Cardiomyopathy and the Infarct Size-Limiting Effect of Ischemic Preconditioning in a Rat Model of Chronic Kidney Disease", Szegedi Tudomanyegyetem (Hungary), 2023

40 13 words / < 1% match - Internet from 06-Dec-2022 12:00AM
archipel.uqam.ca

41 13 words / < 1% match - Internet from 23-Mar-2022 12:00AM
wprim.whocc.org.cn

42 12 words / < 1% match - Crossref
"EUROANAESTHESIA 2005: Annual Meeting of the European Society of Anaesthesiology Vienna, Austria, May 28–31, 2005", European Journal of Anaesthesiology, 09/14/2005

43 12 words / < 1% match - Internet from 31-Oct-2010 12:00AM
content.onlinejacc.org

44 12 words / < 1% match - Internet from 13-Jan-2022 12:00AM
cyberleninka.org

45 12 words / < 1% match - from 18-Dec-2023 12:00AM
kclpure.kcl.ac.uk

46 12 words / < 1% match - from 04-Aug-2023 12:00AM
www.dovepress.com

47 11 words / < 1% match - Crossref
Sohei Arase, Yohei Watanabe, Hiromi Setoyama, Noriko Nagaoka, Mitsuhisa Kawai, Satoshi Matsumoto. "Disturbance in the Mucosa-Associated Commensal Bacteria Is Associated with the Exacerbation of Chronic Colitis by Repeated Psychological Stress; Is That the New Target of Probiotics?", PLOS ONE, 2016

48 11 words / < 1% match - Internet from 10-Nov-2022 12:00AM
assets.researchsquare.com

49 11 words / < 1% match - Internet from 20-Dec-2019 12:00AM
biomedres.us

50 11 words / < 1% match - Internet from 22-Oct-2022 12:00AM
downloads.hindawi.com

51 11 words / < 1% match - from 04-May-2023 12:00AM
www.medrxiv.org

52 10 words / < 1% match - Internet from 14-Jul-2022 12:00AM
docksci.com

53 10 words / < 1% match - Internet from 05-Aug-2019 12:00AM
tessera.spandidos-publications.com

54 9 words / < 1% match - Crossref
A. Bayrak. "Fatty Acid Compositions of Linseed (<i>Linum Usitatissimum</i> L.) Genotypes of Different Origin Cultivated in Turkey", Biotechnology & Biotechnological Equipment, 05/01/2010

55 9 words / < 1% match - Crossref
Ahmed Daak, Adrian Rabinowicz, Kebreab Ghebremeskel. "Omega-3 fatty acids are a potential therapy for patients with sickle cell disease", Nature Reviews Disease Primers, 2018

56 9 words / < 1% match - Crossref
Angel Mojarro, Xingqian Cui, Xiaowen Zhang, Adam B. Jost, Kristin D. Bergmann, Jakob Vinther, Roger E. Summons. "Comparative soft-tissue preservation in Holocene-age capelin concretions", Geobiology, 2021

57 9 words / < 1% match - Crossref
Fatin Atrooz, Karim A. Alkadhi, Samina Salim. "Understanding stress: Insights from rodent models", Current Research in Neurobiology, 2021

58 9 words / < 1% match - Crossref
Tolosa, I., S. Fiorini, B. Gasser, J. Martín, and J. C. Miquel. "Carbon sources in suspended particles and surface sediments from the Beaufort Sea revealed by molecular lipid biomarkers and compound-specific isotope analysis", Biogeosciences, 2013.

59 9 words / < 1% match - Internet from 20-May-2016 12:00AM
shareok.org

60 8 words / < 1% match - Crossref
Hongxiang Guan, Dong Feng, Daniel Birgel, Steffen Kiel, Jörn Peckmann, Sanzhong Li, Jun Tao. "Lipid Biomarker Patterns Reflect Nutritional Strategies of Seep-Dwelling Bivalves From the South China Sea", Frontiers in Marine Science, 2022

61 8 words / < 1% match - Crossref
Ingrid Blixt, Margareta Johansson, Ingegerd Hildingsson, Zoi Papoutsi, Christine Rubertsson. "Women's advice to healthcare professionals regarding breastfeeding: "offer sensitive individualized breastfeeding support"- an interview study", International Breastfeeding Journal, 2019

62 8 words / < 1% match - from 16-Mar-2023 12:00AM
academicjournals.org

63 8 words / < 1% match - from 04-Aug-2023 12:00AM
advances.umw.edu.pl

64 8 words / < 1% match - from 20-Dec-2023 12:00AM
cmjournal.biomedcentral.com

65 8 words / < 1% match - from 29-Jan-2024 12:00AM
pubmed.ncbi.nlm.nih.gov

66 7 words / < 1% match - Crossref
Giovanni Davì, Angela Falco, Carlo Patrono. "Determinants of F2-isoprostane biosynthesis and inhibition in man", Chemistry and Physics of Lipids, 2004
